# Cytomegalovirus Infection Leads to Development of High Frequencies of Cytotoxic Virus-Specific CD4+ T Cells Targeted to Vascular Endothelium

**DOI:** 10.1371/journal.ppat.1005832

**Published:** 2016-09-08

**Authors:** Annette Pachnio, Miriam Ciaurriz, Jusnara Begum, Neeraj Lal, Jianmin Zuo, Andrew Beggs, Paul Moss

**Affiliations:** 1 University of Birmingham, College of Medical and Dental Sciences, Institute of Immunology and Immunotherapy, Edgbaston, Birmingham, United Kingdom; 2 Oncohematology Research Group, Navarrabiomed-Fundación Miguel Servet, IDISNA (Navarra’s Health Research Institute), Pamplona, Spain; 3 University of Birmingham, College of Medical and Dental Sciences, Institute of Cancer and Genomic Sciences, Edgbaston, Birmingham, United Kingdom; University of Wisconsin-Madison, UNITED STATES

## Abstract

Cytomegalovirus (CMV) infection elicits a very strong and sustained intravascular T cell immune response which may contribute towards development of accelerated immune senescence and vascular disease in older people. Virus-specific CD8+ T cell responses have been investigated extensively through the use of HLA-peptide tetramers but much less is known regarding CMV-specific CD4+ T cells. We used a range of HLA class II-peptide tetramers to investigate the phenotypic and transcriptional profile of CMV-specific CD4+ T cells within healthy donors. We show that such cells comprise an average of 0.45% of the CD4+ T cell pool and can reach up to 24% in some individuals (range 0.01–24%). CMV-specific CD4+ T cells display a highly differentiated effector memory phenotype and express a range of cytokines, dominated by dual TNF-α and IFN-γ expression, although substantial populations which express IL-4 were seen in some donors. Microarray analysis and phenotypic expression revealed a profile of unique features. These include the expression of CX3CR1, which would direct cells towards fractalkine on activated endothelium, and the β2-adrenergic receptor, which could permit rapid response to stress. CMV-specific CD4+ T cells display an intense cytotoxic profile with high level expression of granzyme B and perforin, a pattern which increases further during aging. In addition CMV-specific CD4+ T cells demonstrate strong cytotoxic activity against antigen-loaded target cells when isolated directly *ex vivo*. PD-1 expression is present on 47% of cells but both the intensity and distribution of the inhibitory receptor is reduced in older people. These findings reveal the marked accumulation and unique phenotype of CMV-specific CD4+ T cells and indicate how such T cells may contribute to the vascular complications associated with CMV in older people.

## Author Summary

Cytomegalovirus (CMV) is a member of the herpesvirus family and most humans carry chronic CMV infection. This drives the development of large expansions of CD8+ CMV-specific T cells, which increase further during ageing. CMV infection is associated with vascular disease and increased risk of mortality in older people, which may be related to damage from this CMV-specific immune response. Here we used a set of novel reagents called HLA class II tetramers to make a detailed study of CMV-specific CD4+ T cells. We show that CMV-specific CD4+ T cells are found at remarkably high frequencies within blood, representing up to a quarter of all such white cells. In addition they demonstrate a range of unique features. Firstly they carry a chemokine receptor that directs the cells to activated endothelial cells within blood vessels. Secondly, they express epinephrine receptors which would allow them to respond rapidly to stress. Finally, these CD4+ T cells are unique as they are strongly cytotoxic and equipped with the ability to directly kill virally-infected cells. HLA class II tetramers therefore reveal a profile of unique features which provide insight into how CMV-specific CD4+ T cells may be involved in vascular immunopathology.

## Introduction

The effective control of infectious agents requires a range of different arms of the immune system. CD4+ T cells play a pivotal role in orchestrating these events, including support for both antibody production and the expansion and effector function of CD8+ T cells. However it is now well established that CD4+ T cells can also exert crucial effector functions which may be mediated by cytokine production or direct cytotoxicity [1–4]. In chronic viral infections such as cytomegalovirus (CMV) these effector functions are important for control of lytic replication and suppression of viral reactivation. Human leukocyte antigen (HLA) class I tetramers have made a huge contribution to the study of antigen-specific CD8+ T cell immune responses through their ability to allow the visualisation and phenotypic analysis of cells isolated directly from blood and tissue [5]. In contrast, the study of antigen-specific CD4+ T cells has been limited by the relative lack of HLA class II tetramers. Although virus-specific CD4+ T cells can be detected relatively easily by their functional response following exposure to antigen, this alters their phenotype and transcriptome and does not permit analysis of the resting cellular profile. As such, much less is known about the profile and function of antigen-specific CD4+ T cells.

CMV is a β-herpesvirus that establishes a lifelong chronic infection and which is well controlled in healthy people. Initial infection leads to the expansion of very large numbers of virus-specific T cells within peripheral blood which are maintained throughout life and increase even further with age [6–8]. The virus has evolved multiple mechanisms to evade HLA class I and class II-restricted T cell immune responses and a state of functional latency is established after infection, which is associated with intermittent episodes of viral replication (reviewed in [9,10]). HLA class I tetramers have revolutionized the study of the CMV-specific CD8+ immune response and have been pivotal in defining the unique immunodominance of the virus, the phenotype of virus-specific cells and unique aspects of their transcriptome [11]. CMV-specific CD4+ T cells are also critical effector populations in the control of CMV infection where they maintain function of virus-specific CD8+ T cells and suppress viral replication at specific tissue sites [12–17]. Indeed, delayed reconstitution of CMV-specific CD4+ T cells correlates with viral reactivation and CMV disease in organ transplant recipients and is associated with prolonged urinary viral shedding in children undergoing primary infection [18]. In relation to murine CMV, mice lacking CD4+ T cells are impaired in their ability to clear virus from the salivary gland which is an important site of viral latency [19,20].

CMV-specific CD4+ T cells have been identified *in vitro* using cell culture and epitope screening technology. Indeed, the use of peptide pools spanning the whole viral proteome has shown a very broad and strong CD4+ T cell response against many viral proteins of which the most immunodominant are glycoprotein B (gB) and the major tegument component phosphoprotein 65 (pp65) [21]. These studies have shown that the CMV-specific CD4+ T cell response is of unusually strong magnitude and increases further during ageing [15,22–24]. However, such analyses have relied on the interrogation of cells that have been stimulated with antigen for several hours prior to analysis and the almost complete absence of HLA class II tetramers has greatly limited the ability to determine the profile of virus-specific CD4+ T cells directly *ex vivo*.

HLA class II tetramers have recently been used to identify T cells recognising influenza [25,26], hepatitis C virus [27,28], HIV [29] and Epstein-Barr virus [30]. Here we have used three HLA class II tetramers to carry out the first comprehensive analysis of the phenotypic and transcriptional profile of unmanipulated CMV-specific CD4+ T cells. We show that CMV-specific CD4+ T cells are found at very high frequencies within peripheral blood, exhibit a highly differentiated and cytotoxic phenotype which would target them to activated endothelium through CX3CR1-fractalkine binding. These features reveal the extraordinary magnitude of the CMV-specific CD4+ T cell pool that must be maintained to suppress viral reactivation and indicate potential mechanisms that may underlie the development of vascular disease during chronic CMV infection.

## Results

### HLA class II tetramers identify expanded populations of CMV-specific CD4+ T cells during chronic infection of healthy donors

Glycoprotein B and pp65 are the two most immunodominant target proteins for CMV-specific CD4+ T cells. We obtained HLA-peptide tetramers that contained three epitopes derived from gB or pp65, the gB-derived DYSNTHSTRYV peptide restricted by HLA-DRB1*07:01 (DR7) as well as two pp65-derived epitopes, AGILARNLVPMVATV and LLQTGIHVRVSQPSL, which are restricted by HLA-DRB3*02:02 (DR52b) and HLA-DQB1*06:02 (DQ6) respectively (Table 1). These epitopes are subsequently named by the first three amino acids of their respective peptide sequence throughout this paper.

**Table 1:** Details about the HLA class II tetramers used in the study (antigen source, peptide sequence and HLA allele) and the donor cohort.

	DYS:DR7	AGI:DR52b	LLQ:DQ6
antigen	glycoprotein B	pp65	pp65
peptide sequence^a^	DYSNTHSTRYV	AGILARNLVPMVATV	LLQTGIHVRVSQPSL
coordinates	217–227	489–503	41–55
HLA-restriction	DRB1*07:01	DRB3*02:02	DQB1*06:02
number of donors:	33	30	23
CMV-specific T cells detected in:	29 (87.9%)	17 (56.7%)	10 (43.5%)
median frequency (range) [% of CD4+]:	0.66 (0.05–11.75)	0.21 (0.01–2.97)	0.75 (0.09–24.02)
average age in years (range)	50 (25–87)	52 (24–88)	58 (24–87)
male: female	17:16	16:14	8:15
number of donors per age group^b^:			
young: 20-40yrs	11 (11)	7 (3)	3 (2)
middle aged: 41–60 yrs	13 (9)	14 (12)	10 (6)
older adults: over 60 yrs	9 (9)	8 (2)	9 (2)

^a^Epitopes are named referring to the first three amino acids of their peptide sequence (underlined)

^b^in brackets number of donors tetramer+ cells were detected in

**Fig 1:**
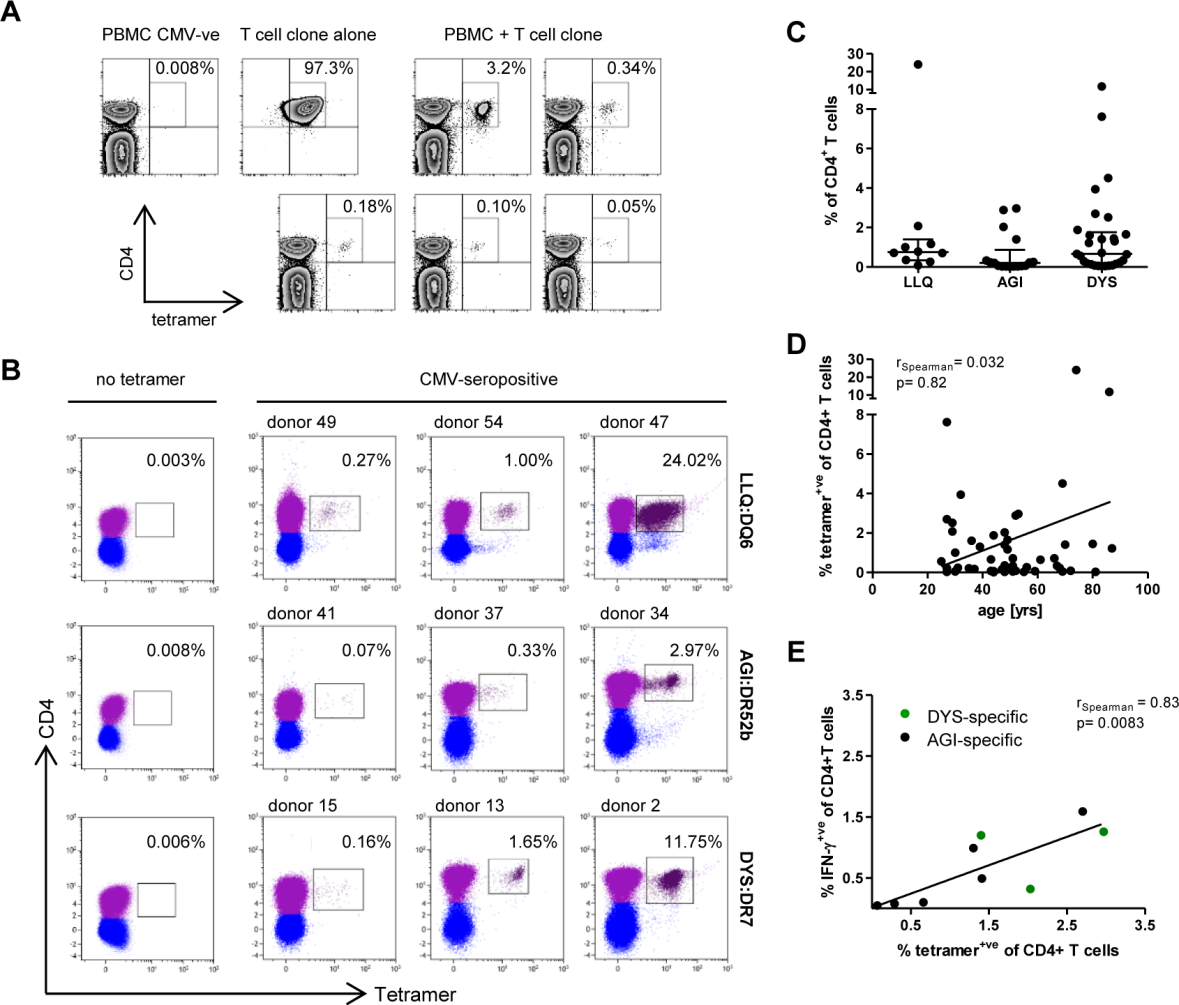
Detection of CMV-specific CD4+ T cells in healthy virus carriers using HLA class II tetramers. (A) Specificity of HLA class II tetramers was tested by staining HLA-matched PBMCs of a CMV seronegative donor and an epitope-specific T cell clone (representative example shown for DYS:DR7). At the same time sensitivity of the tetramer reagents was tested by mixing decreasing numbers of the clonal T cells with PBMCs of the CMV-seronegative donor. Plots are representative of two independent experiments. Similar results were obtained for all three HLA class II tetramers tested. (B) Representative examples of healthy donor PBMCs stained with each of the three HLA-peptide tetramers used in this study. Background staining without tetramer is very low and examples are shown of epitope-specific T cells at low, intermediate and high frequencies. Numbers indicate the proportion of tetramer+ (TM+) cells within the total CD4+ T cell population. (C) Summary of all data for all individuals tested in this study depicting frequency of TM+ cells within the total CD4+ T cell population for each epitope and (D) the correlation between frequency of TM+ cells and donor age (n = 56). (E) The frequency of cells detected by tetramer staining correlates strongly with the proportion of IFN-γ producing cells detected by intracellular cytokine staining following stimulation with the cognate peptide. Spearman‘s rank correlations were performed to measure the strength of associations between variables (D and E).

To confirm the specificity of all three tetramers we initially used the reagents to stain CD4+ T cell clones specific for the cognate HLA class II-peptide complex. This confirmed strong and specific binding whilst very little background was observed following staining of peripheral blood molecular cells (PBMCs) from CMV-seronegative individuals who expressed the appropriate HLA allele contained within each tetramer (Fig 1). The sensitivity of detection of virus-specific T cells through use of tetramer staining was defined by mixing aliquots of peptide-specific T cell clone (5%, 1%, 0.5%, 0.25% and 0.1%) with PBMCs taken from a CMV-seronegative individual. This approach showed that CMV-specific T cells could be identified reliably at frequencies as low as 0.01–0.05% of the total CD4+ T cell population (Fig 1A)

We next went on to use the HLA class II tetramers to enumerate CMV-specific CD4+ T cells within the peripheral blood of healthy donors. PBMCs were isolated from 73 CMV-seropositive individuals between the age of 24 and 88 years who all expressed the appropriate HLA class II allele contained within the tetramer (Table 1). These were then stained directly with the HLA class II tetramer and analysed by flow cytometry (Fig 1B and 1C). CMV-specific CD4+ T cells were observed in 74% of the donors that were tested. The median frequency of CMV-specific CD4+ T cells was 0.45% of the total CD4+ subset, and this varied between 0.75%, 0.21% and 0.66% for T cells specific for the LLQ, AGI and DYS epitopes respectively. The proportion of CD4+ epitope-specific T cells ranged from 0.01% up to a remarkable value of 24% of all CD4+ T cells within one individual. Interestingly, 26% of the donors (19/73) carried peptide-specific T cell populations representing over 1% of the total CD4+ T cell pool.

An increase in the number of CMV-specific CD8+ T cells in association with aging, sometimes termed ‘memory inflation’, has been demonstrated through the use of HLA-peptide tetramer staining. We therefore analysed the frequency of virus-specific CD4+ T cells in relation to age (Fig 1D). Although the very largest tetramer-staining populations were indeed identified in the older donors in our study, no clear increase in CMV-specific CD4+ T cells was observed with age as many younger donors also carried substantial frequencies of CMV-specific CD4+ T cells.

CMV-specific T cells have been detected previously by Interferon (IFN)-γ production following antigen stimulation [23,31] and we were interested to compare the relative number of CD4+ T cells identified by HLA class II tetramers compared to this functional response. Analysis of intracellular cytokine staining (ICS) for IFN-γ production after stimulation with CMV peptide was therefore performed within a panel of donors. A strong correlation was observed between these two values (r_Spearman_ = 0.83; p = 0.008; Fig 1E) although it was of interest that the number of cells detected by tetramer staining was greater than the value obtained by cytokine detection. This indicates that both the number of virus-specific cells has been underestimated in previous studies using cytokine detection and that peptide-specific T cells display other functional responses in addition to production of IFN-γ.

### CMV-specific CD4+ T cells display a multifunctional profile

**Fig 2:**
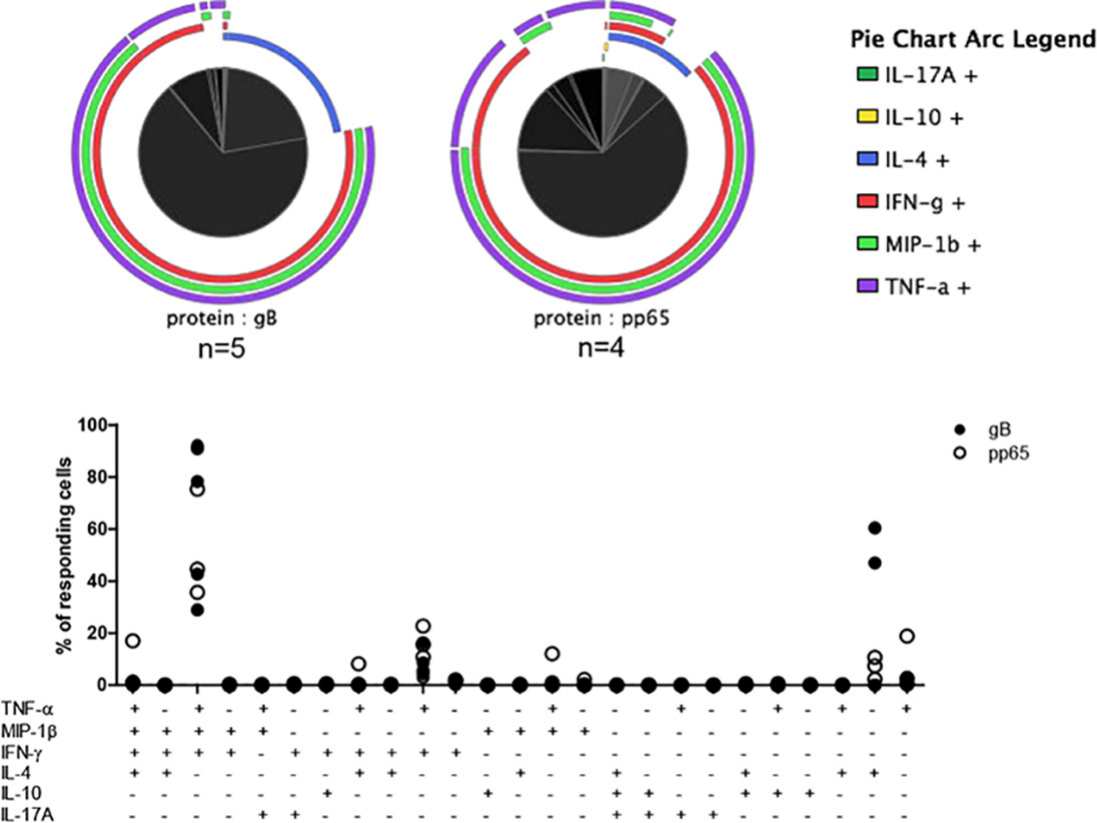
Functional characterisation of CMV-specific CD4+ T cells. Following peptide stimulation of PBMCs the cytokine profile of activated CD4+ T cells was analysed using multicolour flow cytometry. Pie charts in the top summarise the data for gB (DYS)- and pp65-specific T cells (AGI and LLQ), each arc representing one of the cytokines studied, and showing the proportion of responding cells making them. The bottom graph shows individual responses and the proportion of cells detected in relevant different functional subsets.

To further investigate the functional properties of CMV-specific CD4+ T cells following activation, we next went on to stimulate PBMCs with peptide prior to assessment of the profile of cytokine production using ICS. The predominant expression pattern was of combined IFN-γ, TNF-α and MIP-1β (CCL4) production, with a further subset which failed to generate MIP-1β (Fig 2). Of note, the proportion of TNF-α+ cells in most donors exceeded that of IFN-γ+ cells. Interestingly, in three individuals the proportion of IFN-γ+ cells was only between 36–65% and large populations of cells were observed which produced IL-4, usually in isolation and sometimes in combination with other cytokines. Indeed, in one donor these comprised up to 60% of peptide-specific cells. Virtually no CMV-specific CD4+ T cells produced IL-17A or IL-10 in response to antigen stimulation and no significant differences were observed between T cells recognising the gB or pp65-derived epitopes.

### CMV-specific T cells display a unique pattern of CD4+ T cell differentiation

**Fig 3:**
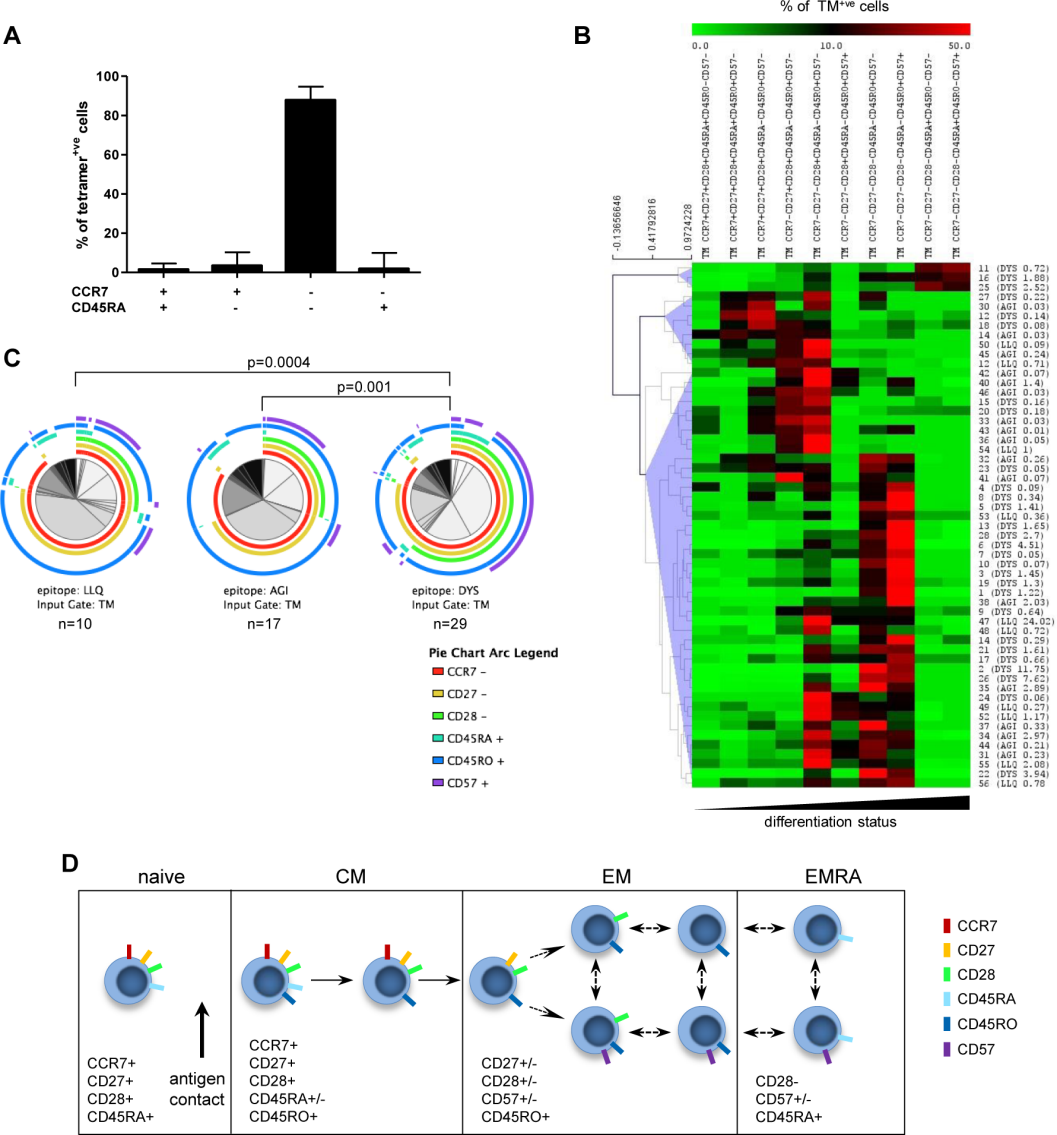
Phenotype of CMV-specific CD4+ T cells. (A) The memory phenotype of virus-specific T cells was determined initially using co-expression patterns of CCR7 and CD45RA (n = 56). Bars represent median with interquartile range. The cells were then further characterised by staining with CCR7, CD27, CD28, CD57, CD45RA and CD45RO. (B) Using Boolean gating all possible combinations of the six surface markers were determined and cells were categorised according to differentiation status, with the least differentiated subset on the left. The heatmap represents data for each individual response, showing the proportion of tetramer positive (TM+) cells within each subset (% of TM+ cells, scale bar at the top). Labelled to the right are the number of the donor and in brackets the peptide epitope and frequency of TM+ cells. (C) We then examined and compared expression patterns between T cells specific for all three CMV-derived epitopes. Each pie chart summarises data for one of the epitopes studied, arcs representing the proportion of TM+ cells expressing any particular marker. To compare T cells specific for the different epitopes (pie charts) permutation analysis (non-parametric test) was performed in SPICE. (D) Proposed stages of differentiation of CMV-specific CD4+ T cells.

We next undertook a more detailed analysis of the phenotype of CMV-specific CD4+ T cells. Expression of CCR7 and CD45RA was used to define naïve, central memory (CM), effector memory (EM) and revertant CD45RA+ effector memory (EMRA) cells. A median of 88% of CMV-specific CD4+ T cells displayed an EM phenotype (CCR7-CD45RA-) with only 3.3% of cells expressing a CCR7+CD45RA- profile typical of CM cells (Fig 3A). In addition, re-expression of CD45RA was found on only a minor subset (1.8%) of effector memory cells, in marked contrast to the profile observed commonly on CMV-specific CD8+ T cells [6]. The surface expression of the additional differentiation markers CD27, CD28, CD57 and CD45RO was then assessed in order to undertake a more detailed phenotypic analysis. A Boolean gating strategy was used to investigate expression patterns of these markers and determine the differentiation hierarchy of the CMV-specific CD4+ T cells (Fig 3B). As anticipated, the majority of the global CD4+ T cell population in each donor expressed a naïve phenotype but CM and EM populations were also observed in comparable proportions (S1 Fig). The differentiation pattern of the CMV-specific T cells was very different (Fig 3B) and this, when combined with current understanding of T cell biology [32,33], allowed us to model the profile of CMV-specific CD4+ T cell differentiation (Fig 3D). This revealed that CMV-specific CD4+ T cells can be detected in several stages of differentiation and reveals progression through a dual CD45RA+CD45RO+ stage prior to loss of CD45RA expression and attainment of CCR7+CD45RO+ central memory phenotype. Further differentiation led to downregulation of CCR7 followed by a sequential loss of CD27 and CD28 expression. Indeed, 64% of cells exhibited a predominant CD27-CD28- profile whereas 22% (12/55) displayed a largely CD27-CD28+ profile. CD57 expression is a predominant feature of CMV-specific T cells and was observed almost exclusively on CD27-CD28- cells, with minor expression on the CD27-CD28+ population. A final stage of differentiation, in a minority of cells, was the re-expression of CD45RA which coincided with complete loss of CD45RO expression.

Further examination of virus-specific populations within individual donors revealed a moderate degree of heterogeneity in relation to differentiation status. Indeed it was noteworthy that 9% (5/55) of responses were characterised by a dominant central memory phenotype whilst only 3 responses exhibited late stage CD45RA+ effector memory (EMRA) differentiation (Fig 3B). As such we next went on to examine potential factors that might be related to the differentiation profile of virus-specific CD4+ T cells. Interestingly, this was not correlated with the magnitude of the immune response, a pattern that is different to the profile for virus-specific CD8+ T cells where clonal expansion is associated with a greater degree of differentiation [6]. In contrast, antigenic specificity may be an important factor as CD4+ T cells specific for DYS (glycoprotein B) displayed a more differentiated phenotype compared to pp65-specific T cells (Fig 3C). As such, loss of CD28 or gain of CD57 expression was seen on 62% and 41% of gB-specific T cells respectively, compared to only 30% and 18% of CD4+ T cells specific for the epitopes from pp65. Moreover, an EMRA phenotype was observed only on the CD4+ T cells which were specific for DYS.

The availability of HLA-peptide tetramers allows the direct analysis of antigen-specific T cells without prior stimulation and this was felt to be particularly valuable in the assessment of CD4+ T regulatory function as FoxP3 expression can be induced following activation through the TCR [34]. In order to investigate whether CMV-specific CD4+ T cells contain natural T regulatory cells we stained cells directly *ex vivo* with HLA class II tetramer, anti-CD4, anti-CD25, anti-CD127 and intracellular anti-FoxP3. However, virtually no CMV-specific T cells were found to exhibit a CD4+CD25+CD127low/-FoxP3+ T regulatory phenotype (S2 Fig).

### Transcriptional analysis of CMV-specific CD4+ T cells reveals high level expression of genes associated with cytotoxicity and chemotaxis

The availability of HLA class II-peptide tetramers allowed us to undertake direct purification and transcriptional analysis of CMV-specific CD4+ T cells, an approach that has been important in relation to determining novel features of the equivalent CD8+ T cell subset [11]. CMV-specific CD4+ T cells were isolated from the blood of five CMV-seropositive donors by staining with tetramer followed by high purity cell sorting. Two of these populations were specific for epitope DYS and three recognised the peptide LLQ. Effector memory T cells isolated from CMV seronegative individuals were used as a comparator group.

**Fig 4:**
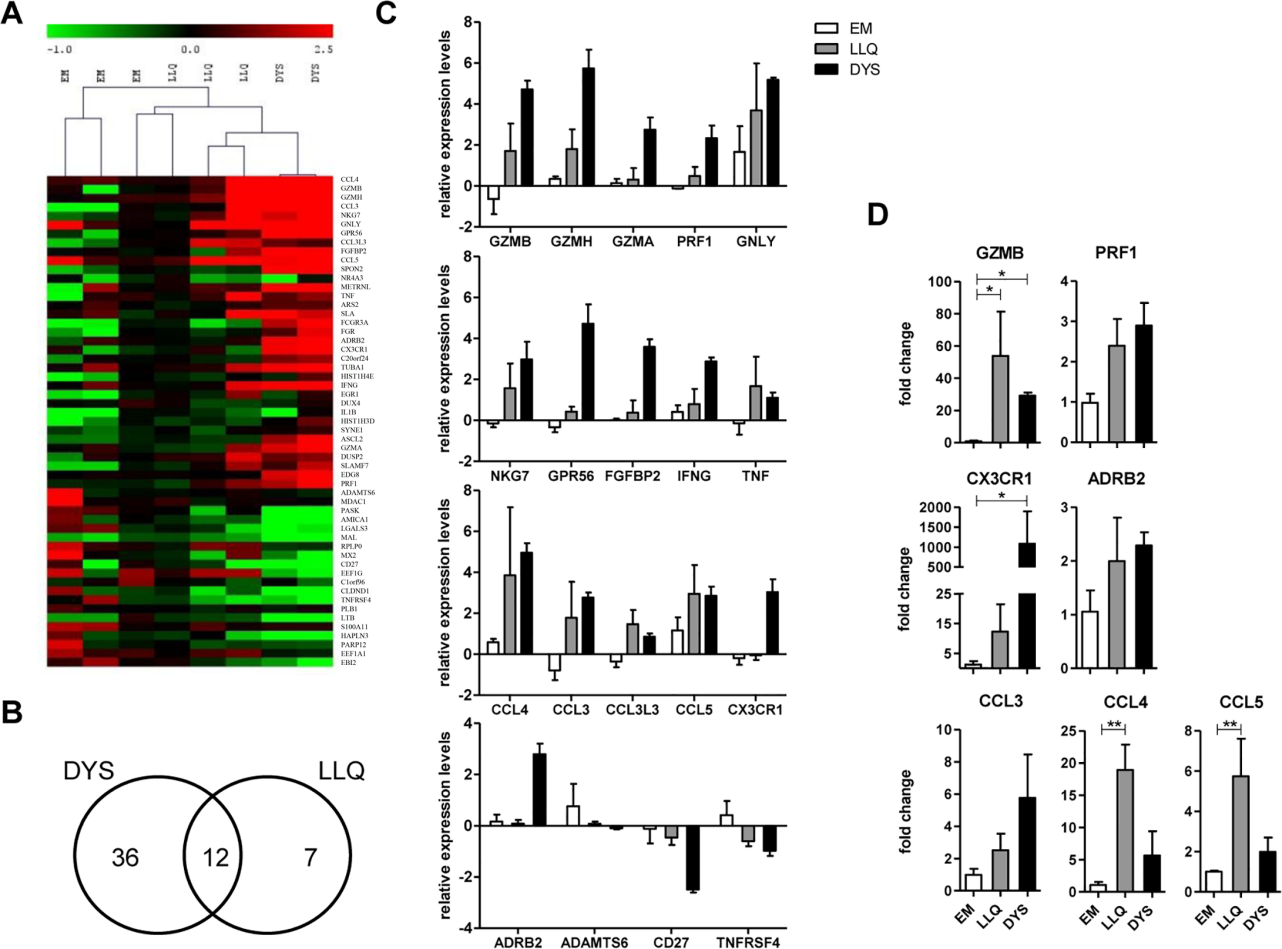
Transcriptional analysis of CMV-specific T cells. (A) CD4+ T cells specific for gB-derived epitope DYS and pp65-derived epitope LLQ were sorted as well as EM CD4+ T cells (CCR7-CD45RA-) of CMV-seronegative individuals and the mRNA transcriptional profile determined using microarray analysis. Transcription of 55 genes was at least 2-fold up or downregulated in CMV-specific T cells compared to the EM cells, which is graphically presented here. (B) The Venn diagram summarises differences observed between EM cells and T cells specific for the two CMV-derived epitopes. (C) Relative transcript levels of aquantile normalised data are depicted for selected genes, comparing EM, LLQ- and DYS-specific T cells. (D) qPCR analysis was performed for genes identified as showing transcriptional upregulation in the microarray analysis. Gene expression levels in TM+ cells were related to those in CD4+ EM cells. Analysis was performed in 2–3 individuals in duplicate. Bars represent means with SEM, Kruskal-Wallis test was performed with Dunn‘s multiple comparison in GraphPad Prism5 to derive p-values.

The pattern of normalised gene expression was compared initially between the combined transcriptome of the CMV-specific T cell samples and the effector memory population from CMV-negative donors. Global expression patterns were broadly similar between the two groups, reflecting the shared effector memory phenotype. However 55 mRNA transcripts differed by at least two-fold expression between the two groups, of which 35 were upregulated in CMV-specific T cells and 20 genes were lower within this group (Fig 4A and S1 Table). We also compared the individual transcriptional profiles of DYS- and LLQ-specific T cell populations and here 12 of the 55 genes that exhibited differential expression between the combined profile of CMV-specific and control EM cells were also differentially expressed in both the DYS- and LLQ-specific T cells. 36 genes were altered only within the DYS-specific populations and 7 genes exhibited differential regulation within LLQ-specific T cells alone (Fig 4B, S2 and S3 Tables), probably reflecting the more marked differentiation profile observed for the DYS-specific population. Relative expression levels (aquantile normalised expression) for selected transcripts are depicted in Fig 4C comparing DYS and LLQ-specific CD4+ T cells, as well as CD4+ EM cells from CMV seronegative donors. An increase in relative transcription levels was often observed for LLQ-specific T cells which was then further enhanced in DYS-specific T cells explaining why more significant differences in gene expression were observed in comparisons between DYS-specific T cells only and EM T cells.

The function of many proteins encoded from the genes upregulated in CMV-specific T cells is related to cytotoxic function, such as granzymes B, H and A, granulysin and perforin. Expression of the chemokines CCL3 (MIP-1α) and CCL4 (MIP-1β) was strongly increased and indicates an important role for CMV-specific CD4+ T cells in attracting cells of the innate immune system to the site of viral recognition. The increased pattern of transcription of CX3CR1 in DYS-specific T cells is of particular note as this chemokine receptor has been shown to be a discriminative marker for CMV-specific CD8+ T cells and is thought to attract cells to areas of stressed endothelium which express the membrane-bound ligand fractalkine [11]. In addition we observed marked overexpression of *ADRB2*, the gene encoding the β2-adrenergic receptor, on these cells which forms an important link between the sympathetic nervous system and the immune system. Additional upregulated genes of interest in CMV-specific T cells included the G protein coupled-receptor GPR56 and fibroblast growth factor-binding protein 2 (FGFBP2), both of which have been previously associated with cytotoxic activity, and the secreted extracellular matrix protein SPON2. As changes in level of transcription do not always translate into the same changes at protein level, further analysis would be needed to confirm some of these observations.

Several genes were downregulated in CMV-specific T cells of which the most striking pattern was seen for *ADAMTS6*, a member of the ADAMTS family (a disintegrin and metalloproteinases with thrombospondin). These secreted proteins have roles in mediating cell adhesion and proteolytic shedding and it is of interest that ADAMTS6 expression is increased by TNF-α [35]. The physiological importance of this will require further investigation as the substrate for ADAMTS6 is currently unknown. CD27 expression was also reduced, reflecting its marked reduction at the cell surface, and levels of *TNFRSF4* (OX40) transcript, which is induced following cell activation, was also low suggesting that CMV-specific CD4+ T cells are largely resting in the steady state.

To further validate results from the microarray analysis, and investigate differences observed between LLQ- and DYS-specific CD4+ T cells, we performed qPCR analysis for genes that were found to exhibit differential expression on microarray. These include the chemokines CCL3, CCL4 and CCL5, GZMB and perforin, as well as CX3CR1 and the ADRB2 gene. For all seven genes we confirmed increased levels of transcription in CMV-specific T cells compared to the CD4+EM population (Fig 4D). The pattern of expression was broadly reflective of that seen in the microarray analysis. Particularly high transcript levels in CMV-specific T cells were observed for GZMB (30 to 50-fold increase), CCL4 (6 to 19-fold increase) and CX3CR1. Of note, in DYS-specific T cells transcription of CX3CR1 was found to be 1000-fold higher than in EM cells and levels were also 12-fold higher in LLQ-specific T cells. Gene expression of ADRB2 was also increased in both LLQ- and DYS-specific T cells.

### CMV-specific CD4+ T cells demonstrate strong cytotoxic potential, high level expression of CX3CR1 and are capable of direct killing of target cells

**Fig 5:**
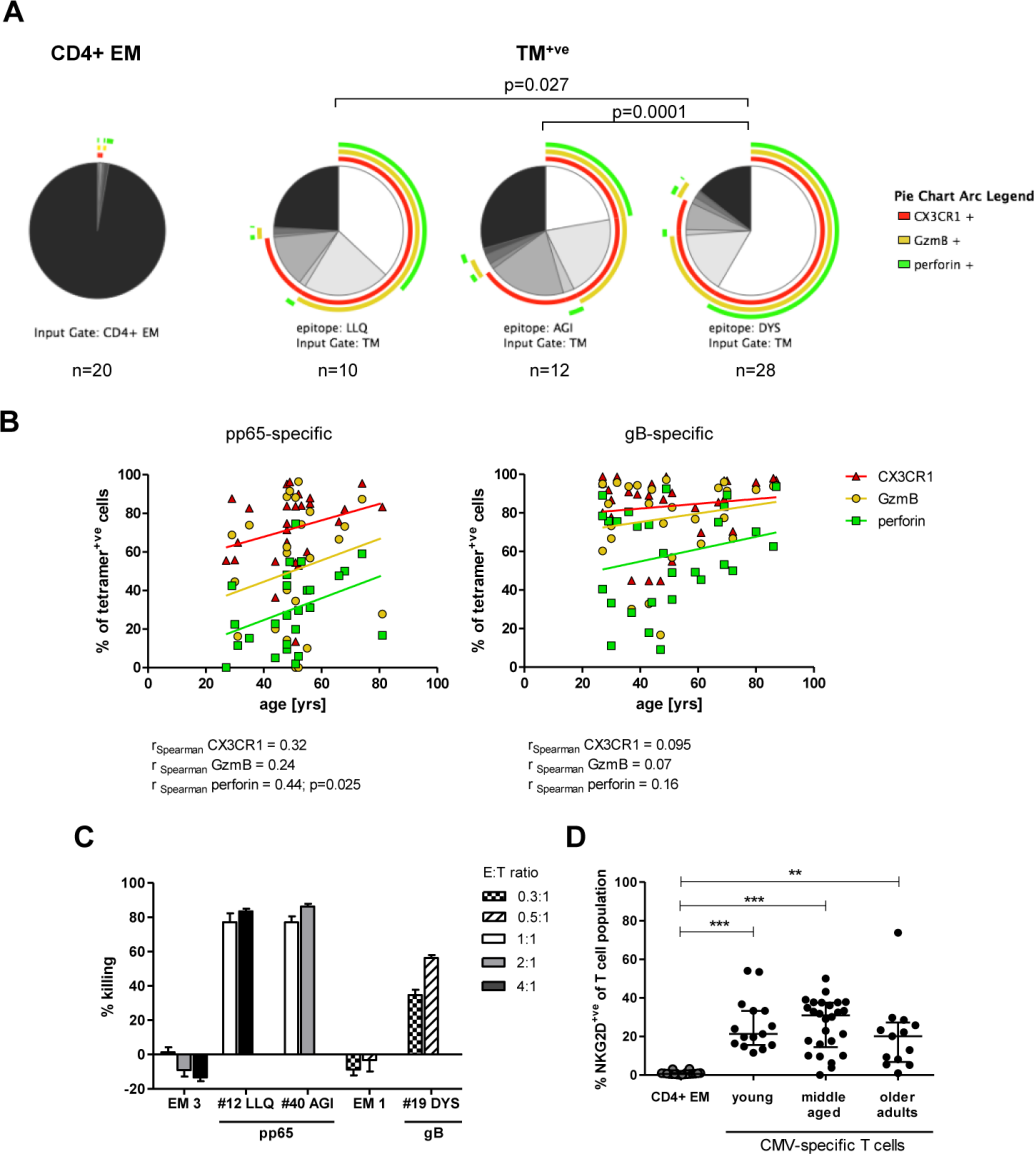
CMV-specific CD4+ T cells are highly cytotoxic directly *ex vivo*. PBMCs were stained with LIVE/DEAD fixable dye, before staining with HLA class II tetramer followed by anti-CD3, CD4 and CX3CR1. After fixation and permeabilisation Granzyme B and Perforin were stained intracellularly. (A) Pie charts represent the proportion of CD4+ EM T cells (in CMV seronegative individuals) or the TM+ population expressing the indicated markers and combinations thereof. To compare pie charts permutation analysis was performed in SPICE. (B) Expression of Granzyme B, perforin and CX3CR1 on pp65-specific (on the left) and gB-specific (on the right) T cells in relation to donor age. Spearman‘s rank correlation was used to analyse the strength of associations between variables. (C) Percentage killing of peptide-loaded target cells (HLA-matched LCLs) mediated by *ex vivo* separated CD4+TM+ or CD4+EM T cells following over night co-culture at effector:target (E:T) ratios indicated. All conditions were performed in triplicate; bars represent means with SEM. (D) Proportion of cells expressing NKG2D within the CD4+ EM or the CMV-specific T cell population in young, middle aged or older adults. Error bars represent medians and IQR. p-values: ** p = 0.001–0.01, *** p = 0.001–0.0001. Kruskal-Wallis test was performed with Dunn‘s multiple comparison in GraphPad Prism5 to derive p-values.

We next went on to investigate the protein expression of four genes whose transcription had been revealed to be strongly upregulated by microarray analysis. As such, tetramer staining was combined with antibodies to granzyme B, perforin, FasL and CX3CR1. CMV-specific CD4+ T cells were found to possess a very strong cytotoxic phenotype with up to 96% of cells staining positive for granzyme B on direct *ex vivo* analysis (Fig 5A). This pattern was particularly strong for glycoprotein B-derived DYS-specific T cells which exhibited a median expression level of 78%, compared to 61% and 45% of the pp65-derived LLQ- and AGI-specific T cells respectively. Perforin expression was also found on 57%, 39% and 27% of these three populations respectively and all T cells that expressed perforin showed co-expression of granzyme B. A very strong correlation was observed in relation to the expression of CX3CR1 with cells of a cytotoxic phenotype (Fig 5A). In CMV seronegative individuals the proportion of CD4+ EM T cells expressing markers of cytotoxicity was only around 1%.

Given the clinical importance of CMV infection in older people we further analysed the expression of granzyme B, perforin and CX3CR1 in relation to the age of the donor (Fig 5B). Interestingly, the substantial cytotoxic potential of CMV-specific CD4+ T cells was found to increase even further with aging and this was particularly the case for pp65-specific T cells, within which perforin expression increased from 18% within donors aged 20–35 years compared to 43% in those aged over 60 years. The cytotoxic profile of DYS-specific T cells also tends to increase with age but this effect was less marked as the cytotoxic phenotype was already strongly established in these cells at an early age. The expression of CX3CR1 was consistently high on CMV-specific T cells from donors at all age groups with 80–90% of both gB and pp65-specific cells carrying this receptor. Expression of FasL was detected on only a very small proportion of CD4+ T cells, with a median of 0.7% of CMV-specific cells and 0.48% of the global effector memory CD4+ pool expressing this marker (S3 Fig).

To investigate whether CMV-specific CD4+ T cells are indeed capable of killing target cells directly *ex vivo*, we isolated virus-specific cells from peripheral blood using tetramers and determined lysis of autologous or HLA-matched lymphoblastoid cell lines (LCLs) loaded with cognate peptide. CD4+ effector memory T cells (CCR7-CD45RA-) from two CMV-seronegative individuals were selected as controls. CMV-specific CD4+ T cells displayed remarkable cytotoxic capacity and this was seen for cells specific for all three peptide targets (Fig 5C). At an effector:target (E:T) ratio of 1:1 LLQ- and AGI-specific T cells lysed around 77% of target cells within 20 hours. DYS-specific T cells were able to kill 56% of target cells even at an E:T ratio of 0.5:1. No killing was observed by CD4+EM cells isolated from CMV seronegative individuals.

Previous studies have shown that CMV-specific CD4+T cells can express the co-stimulatory molecule NKG2D [36] which is almost always present on cytotoxic NK cells and CD8+ T cells. However these analyses relied on functional activation with viral antigen and we therefore examined NKG2D expression on unmanipulated cells through the use of HLA class II tetramers. Interestingly, NKG2D expression was negligible on the CD4+ EM T cell population in CMV seronegative individuals, with a median expression of only 0.66%, but was observed on 23% of CMV-specific CD4+ T cells (Fig 5D). Importantly, this proportion did not show any increase in relation to aging.

### CMV-specific CD4+ T cells express high levels of the inhibitory molecule PD-1

Relatively little is known about the pattern of expression of inhibitory markers such as PD-1 or Tim-3 on antigen-specific CD4+ T cells. Expression of PD-1 on virus-specific CD8+ T cells has been associated with functional impairment of immune responses against HIV or HCV, both chronic infections in which antigen load may remain high for prolonged periods of time [37,38]. However in healthy individuals PD-1^hi^ CD8+ T cells usually demonstrate an effector memory phenotype and do not necessarily exhibit functional ‘exhaustion’ [39].

**Fig 6:**
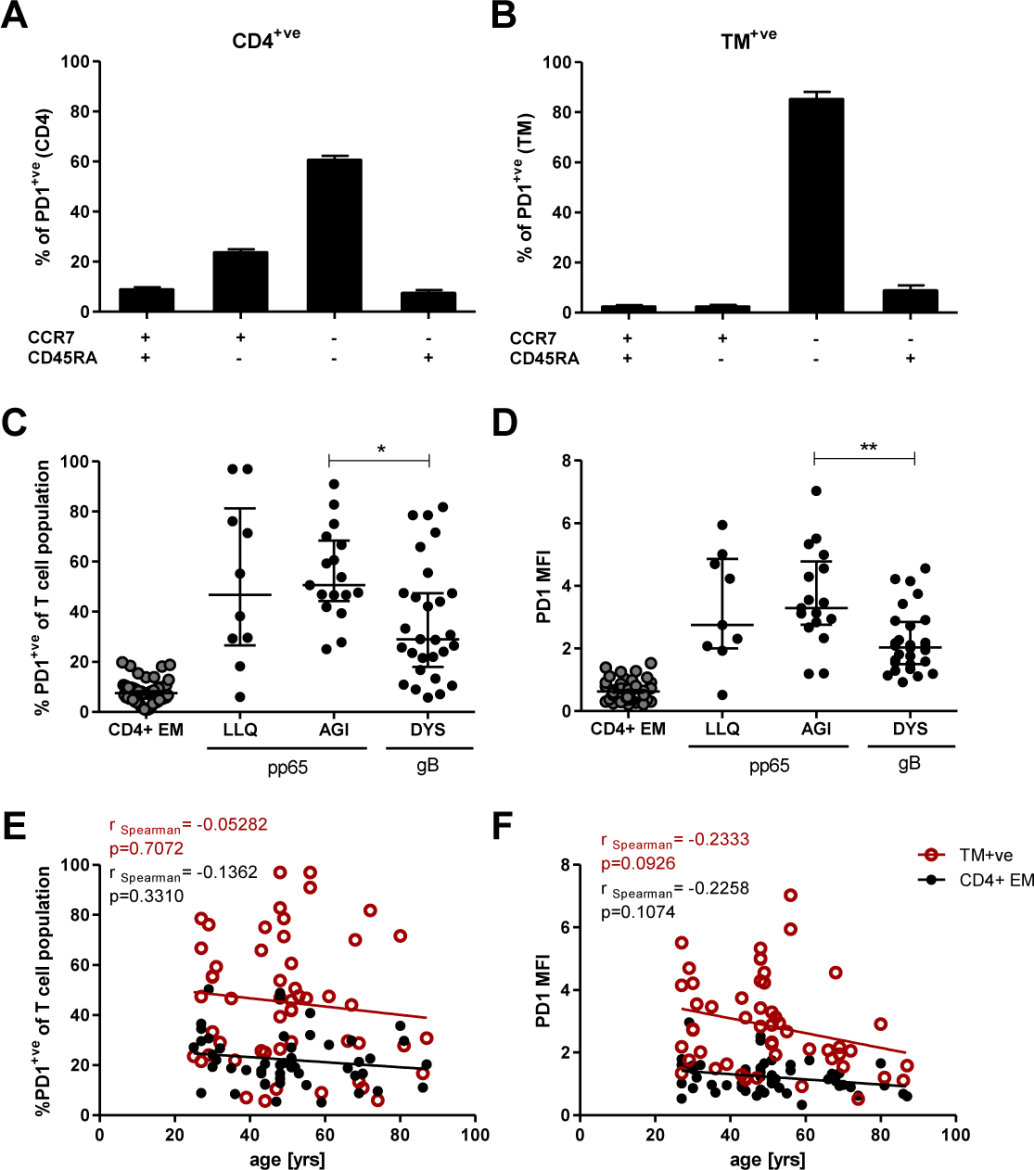
Analysis of inhibitory molecule PD-1 on CMV-specific T cells. PBMCs were stained with LIVE/DEAD fixeable dye, followed by HLA class II tetramer, before staining to detect surface molecules. (A) Percentage of PD-1+ cells within T cell memory subsets defined by co-expression of CCR7 and CD45RA for the total CD4+ population (n = 53). (B) Percentage of PD-1+ cells within those subsets for the TM+ cells (n = 53). Columns represent medians with IQR. (C) The frequency of PD-1+ cells within the CD4+EM T cell subset, LLQ-, AGI- and DYS-specific T cell populations. (D) MFI of PD-1 expression on CD4+EM T cells as well as LLQ-, AGI- and DYS-specific T cells. Error bars indicate medians and IQR. p-values: * p = 0.01–0.05, ** p = 0.001–0.01. Kruskal-Wallis test was performed with Dunn‘s multiple comparison in GraphPad Prism5 to derive p-values (C and D). (E) The proportion of TM+ cells and CD4+EM cells expressing PD-1 in relation to donor age. (F) Median fluorescence intensity (MFI) of PD-1 expression on TM+ cells and CD4+EM cells correlated with donor age. Spearman‘s rank correlation was used to analyse the strength of associations between variables (D and E).

To get a better understanding of PD-1 expression on CD4+ T cell populations we initially analysed the memory phenotype of PD-1+ cells within the global CD4+ T cell subset and the CMV-specific T cell population. This showed that within the CD4+ T cell population 62% of PD-1+ cells carried an EM phenotype and 22% were CM cells (Fig 6A). Of the PD-1+ CMV-specific CD4+ T cells 94% displayed a CCR7-CD45RA- EM phenotype and virtually no cells had a CCR7+CD45RA-CM phenotype (Fig 6B).

We therefore examined the pattern of expression of PD-1 on CMV-specific CD4+ T cells and contrasted this with the pattern of staining on the CD4+EM T cell population. PD-1 expression was observed on a median 47% of CMV-specific CD4+ T cells and exhibited a remarkable range of expression, with cells from some donors showing hardly any evidence of PD-1 expression whereas virtually 100% of cells were positive in other individuals (Fig 6C). When PD-1 expression was evaluated in relation to peptide specificity it was clear that PD-1 expression was markedly reduced on DYS-specific CD4+ T cells, where it was observed on 29% of virus-specific cells, compared to 51% and 47% of AGI- or LLQ-specific T cells, respectively. Interestingly, PD-1 expression was very low on the CD4+EM T cell population and was observed on less than 10% of cells (Fig 6C). When analysing the median fluorescence intensity (MFI) of PD-1 on these cell populations we found the same trends within virus-specific T cells, and much lower MFI values were detected on CD4+EM T cells (Fig 6D). Of interest, Tim-3 expression was not detected on a significant proportion of any CD4+ T cells (S4 Fig).

It might be anticipated that the proportion of T cells expressing an inhibitory marker would increase with age. Therefore we examined both the percentage of PD-1+ cells and the MFI of PD-1 expression on CMV-specific T cells, and the CD4+EM T cell subset, in relation to age. The proportion of PD-1+ cells did not change in relation to the age of the donor (Fig 6E) however a non-significant trend was observed towards lower levels of PD-1 protein expression (MFI) on the surface of PD-1+ CMV-specific CD4+ T cells in older adults (Fig 6F).

## Discussion

In this study we have used HLA class II-peptide tetramers to identify and characterise CMV-specific CD4+ T cells, without the need for functional identification, for the first time. This allowed a comprehensive analysis of the resting phenotype and transcriptional profile of antigen-specific CD4+ T cells recognising glycoprotein B and pp65, the two viral proteins recognised most frequently by CMV-specific CD4+ T cells [21]. The CD4+ T cell response against cytomegalovirus is of interest for several reasons, related primarily to the unusual magnitude and phenotype of virus-specific cells, and also their potential role in the vascular damage that is reported in association with CMV infection in older people [40,41]. Here we identified CMV epitope-specific responses ranging from 0.01% up to a remarkable 24% of the total CD4+ repertoire. The median peptide-specific response was 0.45% of the CD4+ T cell repertoire which is considerably higher than CD4+ T cell responses against viruses such as influenza, hepatitis C Virus and Epstein-Barr Virus [30,42,43].

Interestingly, the frequency of cells identified by tetrameric staining was greater than that detected by expression of IFN-γ following peptide stimulation. As such, although this confirms the strong Th1 profile of CMV-specific CD4+ T cells, it also reveals that a considerable proportion of peptide-specific CD4+ T cells demonstrate an alternative cytokine profile. Indeed, TNF-α was the cytokine most frequently produced by CMV-specific CD4+ T cells. In addition, a proportion of virus-specific T cells was found to secrete IL-4 in some donors although the great majority of CMV-specific CD4+ T cells display a polarized Th1 phenotype [44]. IL-4 production by CMV-specific CD4+ T cells has previously been reported through ELISPOT analysis [45]. Of interest, although IL-4 production within pp65-specific T cells was usually observed with co-expression with IFN-γ, IL-4 production by gB-specific cells was seen in the absence of other cytokines. As such these observations indicate novel functional roles for IL-4 in the setting of chronic infection which warrant further investigation [46]. Functional assays using stimulation with whole viral lysate have demonstrated an increase in virus-specific CD4+ T cells with age [22] and although a similar trend was observed in our analysis it is likely that demonstration of such T cell ‘memory inflation’ will require analysis of responses directed against a wide range of different epitopes. Many younger donors already had high frequencies of epitope specific T cells in the periphery. This may be a reflection of the duration of viral carriage as some individuals acquire the virus very early in life whereas others only do so at a later time.

Almost all CMV-specific CD4+ T cells expressed an effector memory phenotype, although in contrast to CMV-specific CD8+ T cells, very few had reverted to re-expression of the CD45RA isoform rather than CD45RO. *In vitro* studies suggest that re-expression of CD45RA occurs during prolonged absence of antigenic stimulation [47] which may indicate that CMV-specific CD4+ T cells undergo antigen recognition *in vivo* more frequently than CD8+ subsets. Latent CMV is maintained within cells of the monocytic lineage and viral reactivation occurs during maturation into dendritic cells [48]. As such, CMV-specific CD4+ T cells are likely to undergo regular antigenic stimulation and this may serve to retain expression of the CD45RO isoform. Tetramer staining also allowed a detailed analysis of the membrane phenotype of CMV-specific CD4+ T cells and indicated that CD27 expression is lost early during differentiation and is then followed by loss of CD28 in the majority of the population. Loss of CD28 expression on CD4+ T cells is very unusual and, indeed, the CD4+CD28- phenotype is virtually unique to CMV-specific T cells [49] and indicates that alternative mechanisms of T cell co-stimulation become important in the CMV-specific immune response, potentially through molecules such as 4-1BB [50]. CD57 is a poorly characterised molecule, but is again highly specific for CMV-specific T cells and was found to be expressed reciprocally with CD28, suggesting that it may itself have a potential role in co-stimulation. NKG2D is one such potential alternative costimulatory molecule and can synergise with TCR-dependent activation of CMV-specific CD4+ T cells to enhance a range of effector functions [36,51,52]. Indeed, we found that NKG2D was expressed on 23% of CMV-specific CD4+ T cells, a remarkably high level for a molecule typically associated with expression on NK and CD8+ T cells. The high degree of differentiation seen for CMV-specific CD4+ T cells was largely independent of the frequency of epitope-specific T cells, indicating that alternative factors, such as the environment in which T cell priming occurs or the availability of antigen, may influence their phenotypic profile (reviewed in [53]). However the antigenic specificity of virus-specific CD4+ T cells did have a marked influence on the differentiation status such that CD4+ T cells specific for glycoprotein B-derived epitope DYS consistently displayed a more differentiated phenotype than pp65-specific cells. Glycoprotein B is highly unusual in that it has direct access to the HLA class II antigen-processing pathway of infected cells. This mechanism is likely to mediate frequent reactivation of gB-specific T cells and could explain the more ‘driven’ differentiation phenotype of CD4+ T cells specific for peptides from this protein [54,55].

A unique aspect of our study was the direct isolation of virus-specific CD4+ T cells without the need for antigenic stimulation, such that we were able to analyse their resting transcriptional profile. Even though differences to CD4+EM T cells of CMV seronegative individuals were generally modest, the most striking observations were seen in the expression of genes related to cytotoxic function and chemotaxis. Remarkably, the profile of cytolytic genes upregulated in CMV-specific CD4+ T cells closely corresponds to the pattern seen in CD8+ cytotoxic T cells (CTL). These included granzymes A, B and H, as well as perforin, granulysin and NKG7. Importantly, we were also able to determine that Fas ligand is not expressed by virus-specific CD4+ T cells, indicating that the delivery of mediators from cytotoxic granules is the dominant mechanism of target cell lysis. Remarkably, the use of tetrameric staining reveals that this profound cytotoxic potential of CMV-specific T cells is observed within resting cells in the bloodstream. Furthermore isolated virus-specific CD4+ T cells very efficiently kill antigen-loaded target cells directly *ex vivo* suggesting that they are primed for rapid target cell lysis in the event of an episode of viral reactivation.

CMV-specific CD4+ T cells also expressed a distinctive profile of chemokines and additional proteins that indicate an important role in chemotaxis and tissue migration. Indeed, one of the most fascinating features of CMV-specific CD4+ T cells is their high level of expression of CX3CR1, a chemokine receptor that binds CX3CL1 (fractalkine) and has already been identified as a specific marker for CMV-specific CD8+ T cells [11]. The CX3CR1/CX3CL1 axis plays an important role in both the adhesion and transmigration of lymphocytes to endothelial cells during inflammation [56,57]. Interestingly, endothelial cells are a principal target tissue for CMV infection and the expression of CX3CR1 on CMV-specific T cells may therefore act to localise adaptive immunity to sites of viral reactivation. This mechanism of endothelial-targeting may be highly relevant to the potential development of endothelial immunopathology mediated by CMV-specific cytotoxic T cells [58,59]. Indeed a close link has been observed between augmented CMV-specific immune responses and a range of inflammatory conditions (reviewed in [60]) and the proportion of CD4+CD28- T cells has been shown to correlate directly with cardiovascular mortality in some studies [61].

CMV-specific CD4+ T cells also exhibit a very similar chemokine secretion profile to that of virus-specific CD8+ cells, with production of the inflammatory mediators CCL3 (MIP-1α), CCL4 (MIP-1β) and CCL5 (RANTES), all of which would support recruitment of innate immune cells such as macrophages and NK cells. The pattern is associated with high level expression of IFN-γ and TNF-α but very little production of IL-2 [23,62] and is again typical of a differentiated Th1 profile.

An additional interesting observation from the microarray data was the detection of high levels of *ADRB2* mRNA in glycoprotein B-specific T cells. *ADRB2* encodes the β2-adrenergic receptor which allows cells to respond to systemic production of epinephrine and forms an important link between the sympathetic nervous system and the adaptive immune response [63,64]. The CMV genome contains promoter elements that can bind epinephrine leading to increased viral gene transcription [65] and physiological stress is established as a risk factor for reactivation of many herpesviruses. Interestingly, CX3CR1+ T cells have been shown to be the major T cell subset released into the circulation following administration of epinephrine, in a mechanism partially mediated by reversal of their resting adherence to endothelium [66]. This may provide a mechanism of rapid mobilisation for CMV-specific T cells in response to stress induced viral reactivation, but this will need further investigation.

T cell activation and effector function are finely tuned events and CD4+ T cells show an extremely high sensitivity for their cognate antigen [67]. Moreover lytic synapse formation has a very low threshold for activation [68,69]. A proportion of CD27-CD28- CMV-specific CD4+ T cells has previously been described to exhibit regulatory function *in vitro* [70] but FoxP3+ cells were not detectable in this study and antigen-specific T cells were not shown to produce IL-10. This suggests that regulatory mechanisms are likely to be focussed at the level of the effector T cell and here PD-1/PD-L1 interactions play a key role as a negative feedback mechanism controlling TCR-dependent effector function of T cells [71]. High levels of PD-1 have been detected on CD8+ effector memory T cells [39] and similarly we find that PD-1 expression on CD4+ T cells is also largely confined to this subset. PD-1 expression was seen on nearly half of all CMV-specific CD4+ T cells and could serve to limit T cell activation. As such it is noteworthy that the level of membrane PD-1 expression (MFI) on virus-specific CD4+ cells tends to decrease during aging and may therefore lower the activation threshold of T cells at a time when their cytotoxic potential is actually increasing. Together these factors may influence virus-host balance across the life course and may contribute towards immunopathology.

**Fig 7:**
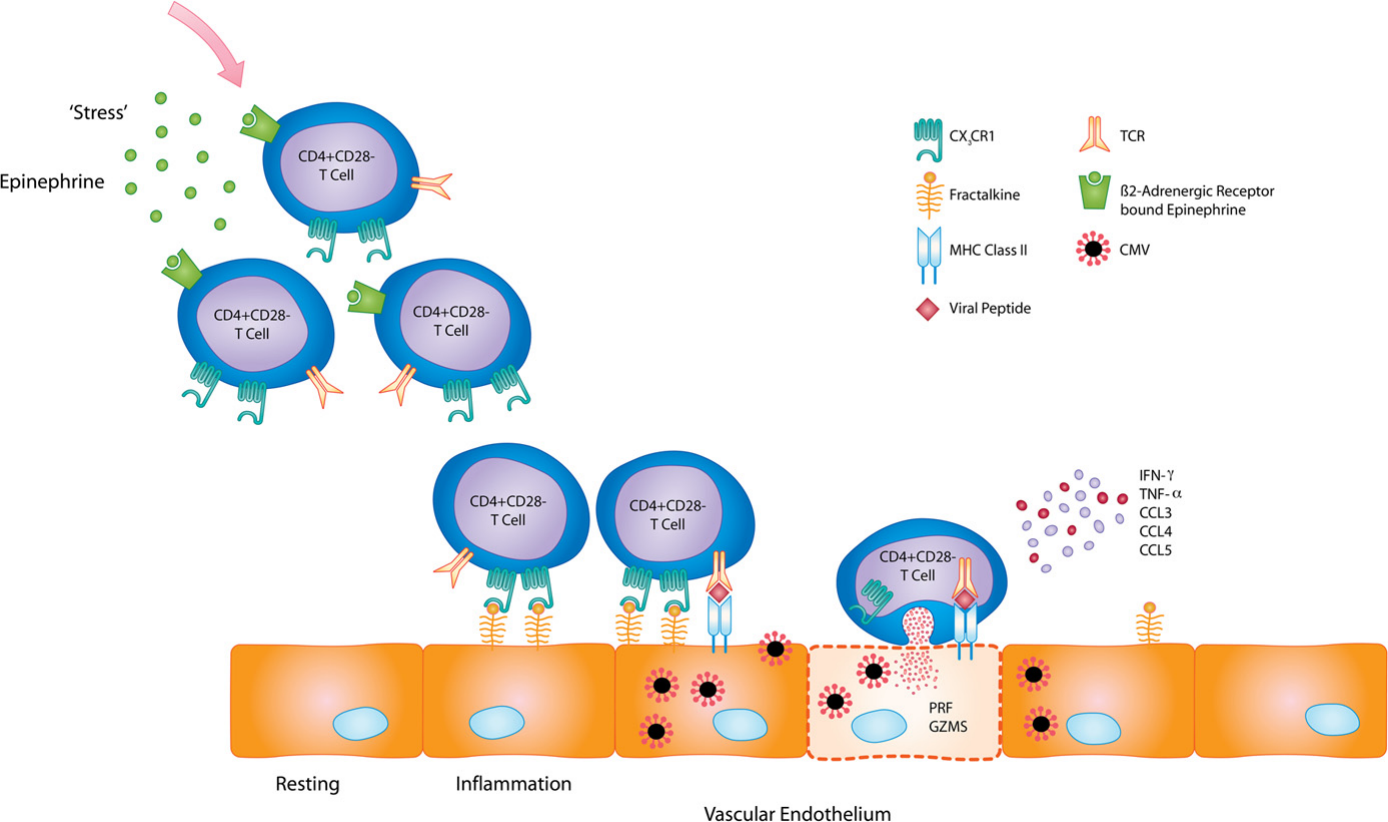
Potential mechanism of virus-directed vascular injury in response to stress and endothelial damage. CMV-specific CD4+ T cells expressing β2-adrenergic receptor would be mobilised into the bloodstream in response to epinephrine released during stress. Epinephrine also leads to reactivation of virus from latently infected endothelial cells. Furthermore, activation/inflammation of endothelium induces expression of membrane-bound fractalkine. CX3CR1+ CMV-specific CD4+ T cells would adhere to endothelium, antigen recognition leading to activation of these highly cytotoxic effector cells and direct killing of virus-infected endothelial cells. Release of cytokines and chemokines from CMV-specific CD4+ T cells potentially attracts further immune cells to such sites of viral reactivation and inflammation which may enhance vascular injury.

In summary our data reveal that people who are chronically infected with cytomegalovirus, which represents the great majority of the global population, harbour substantial populations of virus-specific CD4+ T cells within their bloodstream. These highly differentiated cells display a strongly cytotoxic phenotype, may be targeted to activated endothelium and have the potential to respond to physiological ‘stress’ through detection of epinephrine (Fig 7). In addition, this cytotoxic profile increases further with age whilst the level of inhibitory PD-1 on the surface declines. These findings reveal the exceptional evolutionary adaptation of the CD4+ T cell response towards the control of CMV. In addition they shed light on the potential mechanisms whereby CMV infection may serve to mediate tissue damage, most particularly vascular disease, and indicate a range of potential novel immunotherapeutic targets.

## Materials and Methods

### Ethics statement

The study was approved by the West Midlands (Black Country) Research Ethics Committee (REC 07/Q2702/24) and all donors gave written informed consent before participation. The donor cohort included samples from laboratory personnel, the blood transfusion service and healthy older adults recruited from the ‘Birmingham 1000 Elders group’ (REC 2002/073).

### Study subjects

A total number of 73 CMV-seropositive donors, aged between 24–88 years, with appropriate HLA-genotype were included in the study. PBMCs were isolated from 50ml heparinized blood by density gradient centrifugation using Lympholyte-H cell separation media (Cedarlane) and aliquots of 10x10^6^ cells were cryopreserved in RPMI1640 (Sigma-Aldrich) containing 20% fetal calf serum (FCS) and 10% DMSO and stored in liquid nitrogen until use.

To identify donors with the appropriate HLA-genotype, genomic DNA was isolated from PBMCs using the GenElute Blood Genomic DNA Kit (Sigma-Aldrich) according to manufacturer’s instructions. Typing for HLA class II alleles was performed by PCR technique as described previously [72].

### HLA class II-peptide tetramer complexes

Phycoerythrin (PE)-conjugated custom-made HLA class II tetrameric complexes were purchased from the Benaroya Research Institute at Virginia Mason (Seattle, Washington). Three tetramer complexes were used in this study: They were comprised of the CMV gB-derived epitope DYSNTHSTRYV in the context of HLA-DRB1*07:01 (DR7) [73], or pp65-derived epitopes AGILARNNLVPMVATV within HLA-DRB3*02:02 (DR52b) [74] and LLQTGIHVRVSQPSL within HLA-DQB1*06:02 (DQ6) [75].

Initially, the specificity of each tetramer was confirmed by screening against CD4+ T cell clones recognizing the tetramer’s cognate HLA class II-peptide complex or against PBMCs from a CMV-seronegative donor expressing the appropriate HLA-allele. Optimal tetramer concentration and staining times were distinguished at the outset and constant conditions used throughout the study.

### Cell staining and flow cytometric analysis

#### Surface staining

A total of 1 x 10^6^ PBMCs were stained with LIVE/Dead fixable violet stain (Invitrogen) for 15 minutes at room temperature (RT), followed by a wash step with phosphate-buffered saline (PBS). Cells were then re-suspended in 50 μL of human serum and incubated with PE-conjugated HLA class II tetramer for 1 h at 37°C and 5% CO_2_. After three washes with staining buffer (1xPBS supplemented with 0.5% BSA and 2 mM EDTA), the cells were co-stained for 15 minutes at 4°C with saturating amounts of different combinations of the following antibodies: anti-CD3 AmCyan (SK7, BD Biosciences), anti-CD4 PerCPCy5.5 (RPA-T4, eBioscience), anti-CD4 PE-CF594 (RPA-T4, BD Biosciences), anti-CD4 APC-Cy7 (RPA-T4, Biolegend), anti-CD14 Pacific Blue (HCD14, Biolegend), anti-CD19 eFluor450 (H1B19, eBioscience), anti-CD25 APC-Cy7 (BC96, Biolegend), anti-CD27 APC-eFluor780 (O323, eBioscience), anti-CD28 PE-Cy7 (28.2, Biolegend), anti-CD45RA AF700 (H1 100, Biolegend), anti-CD57 APC (HCD56, Biolegend), anti-CD69 PE-Cy7 (FN50, Biolegend), anti-CD127 PerCP-Cy5.5 (HIL-7R-M21, BD), anti-CCR7 FITC (150503, R&D), anti-CX3CR1 PerCP-Cy5.5 (2A9-1, Biolegend), anti-FasL AF488 (14C2, AbD Serotec), anti-NKG2D PE-CF594 (1D11, BD Biosciences), anti-PD-1 PerCP-Cy5.5 (EH12.2H7, Biolegend) and anti-Tim3 APC (F38-2E2, eBioscience). Following a final wash cells were re-suspended in 200 μL of staining buffer for acquisition.

#### Intracellular staining

For detection of intracellular perforin and granzyme B, cells were stained for surface antigens as described above, then fixed in 4% paraformaldehyde (Sigma-Aldrich) for 15 minutes at RT in the dark. Cells were washed and anti-perforin PE-Cy7 (γG9, eBioscience) and anti-granzyme B AF647 (GB11, Biolegend) antibodies were added in the presence of 0.5% saponin (Sigma-Aldrich) in PBS, incubated for 30 minutes at RT in the dark followed by a washing step and re-suspended in 200 μL of staining buffer.

For detection of intracellular FoxP3, cells were stained with surface markers as above and after a washing step fixed for 30 minutes using 1X Fix/Perm buffer (eBioscience). Cells were washed and then permeabilized in Perm buffer (eBioscience) for 15 minutes in the dark and subsequently stained with anti-FoxP3 AF647 (206D, Biolegend) for 30 minutes at RT in the dark before being washed and re-suspended in 200 μL of staining buffer.

All stained cells were acquired using an LSRII flow cytometer and DIVA software (BD Biosciences) collecting at least 300,000 live lymphocytes and all data was processed using Kaluza 1.3 software (Beckman Coulter). For the analysis single, viable, CD14-CD19- lymphocytes were gated on before selecting CD3+CD4+ T cells and CD3+CD4+tetramer+ cells which were then further analysed for expression of surface and/or intracellular staining (S5 Fig). For multicolour staining panels additionally Boolean gating was used to identify all possible combinations of markers stained for on CD3+CD4+ and CD3+CD4+tetramer+ T cell populations. This data was further analysed using SPICE version 5.3 [76] and MultiExperiment viewer (MeV) version 4.9 [77].

### Peptide stimulation and intracellular cytokine staining

To identify cytokine-producing T cells following activation, 1x10^6^ PBMC were resuspended in RPMI1640 (Sigma-Aldrich) supplemented with 10% FCS and 1% Penicillin/Streptomycin and stimulated with peptide (5μg/ml final concentration) overnight at 37°C with 5% CO_2_ in the presence of BrefeldinA (10μg/ml final concentration; Sigma-Aldrich). Unstimulated cells and cells stimulated with Staphylococcus Enterotoxin B (final concentration 0.2μg/ml; Sigma-Aldrich) served as controls. Following overnight incubation the cells were stained with LIVE/Dead fixable violet or aqua stain (Invitrogen) as described above, T-cells were then identified by staining with anti-CD4-PE (RPA-T4, BD Bioscience) or anti-CD3 AF700 (SK7, Biolegend) and anti-CD4 APC-Cy7 and B cells excluded by staining with anti-CD19 pacific blue (H1B19, eBioscience; dump channel). Fixing was carried out with 4% paraformaldehyde (in PBS; Sigma-Aldrich) for 15 min at RT before permeabilising with 0.5% Saponin (in PBS; Sigma-Aldrich) for 5 min. Intracellular cytokines were then stained with the following antibodies: anti-IFN-γ FITC antibody (25723.11, BD Bioscience) or anti-IFN-γ PE-Dazzle (4S.B3), anti-TNF-α PE-Cy7 (Mab11), anti-IL-10 PE (JES3-9D7), anti-IL-4 BV421(MP4-25D2; all Biolegend), anti-IL17A (eBio64DEC17, eBioscience) and anti-MIP-1β (24006, R&D) and followed by a final wash in staining buffer. Acquisition was carried out on an LSR II flow cytometer and DIVA software (BD Bioscience) collecting 300,000 live lymphocytes and data analysed using FlowJo software version 7.6.5 (Tree Star). For the analysis single, viable, CD19- lymphocytes were gated before identification of cytokine-producing CD4+ T cells. For the multi-cytokine panel Boolean gating was used to determine all possible combinations and further analysis was carried out using SPICE version 5.3.

### Cell separation and microarray analysis

To analyse the transcriptional profile of CMV-specific T cells we sorted DYS- and LLQ-specific CD4+ T cells from CMV-seropositive healthy donors and for comparison CD4+ T cell subsets from CMV-seronegative healthy individuals. For this PBMCs were isolated from 120 mL of heparinised blood by density gradient centrifugation and the CD4+ T cell population was enriched by negative selection (StemCell Technologies) according to manufacturer’s instructions. CD4+ T cells from CMV-seropositive donors were then stained with LIVE/Dead fixable far red stain (Invitrogen), washed and re-suspended in 600 μL of human serum prior to incubation with PE-conjugated HLA class II tetramer (HLA-DR7:DYS or HLA-DQ6:LLQ) for 1 h at 37°C and 5% CO_2_. After washing, cells were stained with anti-CD4 PE-CF594 (RPA-T4, BD) and re-suspended in RPMI + 10% FCS. From the single, viable lymphocyte population CD4+tetramer+ cells were then sorted on a MoFlow Cell Sorter (Beckman Coulter) consistently reaching a purity of 98–99%. Following CD4-enrichment cells of CMV-seronegative donors were stained with LIVE/Dead fixable far red stain (Invitrogen), anti-CD4 PE-CF594 (RPA-T4, BD Biosciences), anti-CCR7 FITC (150503, R&D) and anti-CD45RA PE (HI100, eBioscience). Based on their expression profile effector memory cells (CCR7-CD45RA-) were then sorted to high purity.

Total RNA of the sorted cells was extracted using an RNeasy Plus Mini kit (Qiagen) according to manufacturer’s instruction. The RNA integrity was checked and it was subsequently labelled before hybridisation to Agilent human gene expression 8x60k microarrays (G4858A) according to manufacturer’s instructions following the standard Agilent Low Input Quick Amp labelling protocol. Due to low mRNA yield, CD4+CD45RO+ T cells sorted from a CMV-seronegative donor served as the reference sample on the two-colour microarray slide. These were not taken into account for the analysis. The Microarrays were carried out by the Functional Genomics, Proteomics and Metabolomics Facility at the School of Biosciences, University of Birmingham.

Microarray data was analysed with the R Limma Package (Bioconductor) [78–80]. Normalisation was performed with the Loess (intra-array) and Aquantile (inter-array) methods. An adjusted p-value (Benjamini and Hochberg's method) of 0.05 and below was taken as significant for differences in gene expression or otherwise a 2-fold change in expression levels. Further analysis of output data was completed in Excel (Microsoft Corp). Hierarchical clustering was performed on the MultiExperiment viewer version 4.9 (MeV, [77]. Functional enrichment analysis was completed using DAVID version 6.7 [81,82].

Microarray data are available in the ArrayExpress database (www.ebi.ac.uk/arrayexpress) under accession number E-MTAB-4510.

### Quantitative PCR to validate microarray findings

cDNA generated from the same samples used for the microarray analysis was used to analyse transcription of a selected number of genes that were differentially expressed between CD4+EM cells and CMV-specific CD4+ T cells. TaqMan Gene Expression Assays for CCL3 (Hs04194942_s1), CCL4 (Hs04421399_gH), CCL5 (Hs00982282_m1), GZMB (Hs00188051_m1), PRF1 (Hs00169473_m1), ADRB2 (Hs00240532_s1), CX3CR1 (Hs04187059_m1) and GAPDH (4310884E) were bought from Thermo Fisher Scientific. Specific target amplification was carried out on the cDNA using 2× TaqMan PreAmp Master Mix (Life Technologies) and 0.2× primer mix (20× TaqMan assays diluted in water). Reactions were subjected to 95°C for 10 min, followed by 12 cycles of 95°C for 15 s and 60°C for 4 min. These pre-amplified samples were then diluted 1:5 with water prior to Q-PCR analysis using the TaqMan Gene Expression Assays. The relative transcription was calculated comparing with average transcription level of the three control CD4+EM T cells and GAPDH assays served for normalization. Assays were performed in duplicate for two to three donors each (EM, LLQ and DYS). Transcription levels in CD4+EM T cells were set to 1.

### Killing of target cells by CMV-specific CD4+ T cells

To analyse the cytotoxic capacity of CMV-specific CD4+ T cells directly *ex vivo*, CD4+TM+ cells were separated as described above and then co-cultured over night with CFSE labelled (0.5μM; Invitrogen) autologous or HLA-matched LCLs which were loaded with the cognate peptide. CFSE-labelled LCL alone served as control. In addition CD4+ CCR7-CD45RA- (EM) cells of two CMV seronegative individuals were sorted and served as effector cells. Killing of target cells was assessed on a BD Accuri flow cytometer (BD Biosciences) by quantifying live CFSE-labelled LCL using counterstaining with Propidium Iodide (PI) to identify dead cells. All conditions were carried out in triplicate. For the analysis initially the T cell population and the LCL population were gated on, using FSC and SSC scatter plots, to determine the true ratio of effector to target cells. Then the number of live LCLs was determined using CFSE and PI and percent killing of target cells was calculated.

### Statistical analyses

Statistical analysis of flow cytometry data was performed using GraphPad Prism5. The non-parametric Mann-Whitney *U*-test was applied for comparison of two groups, and the Kruskal-Wallis test (with Dunn’s multiple comparison) for comparison of more than two groups of continuous measurements. To analyse the strength of associations between variables Spearman’s rank test was used. A p-value of less than 0.05 was considered statistically significant.

## Supporting Information

S1 Fig Phenotype of total CD4+ T cell population. Cells were stained with CCR7, CD27, CD28, CD57, CD45RA and CD45RO to characterise the overall phenotypic profile. Using Boolean gating all possible combinations of the six surface markers were determined, and those subsets containing cells categorised according to differentiation status (increasing from left to right) as in Fig 3B. The heatmap is representing the proportion of CD4+ T cells within each subset for each of the donors studied.
(TIF)

S2 Fig Detection of regulatory T cells within CD4+ or TM+ populations. (A) Example scatter plots showing co-expression of FoxP3 and CD25 on CD4+ or TM+ T cells. (B) Frequency of FoxP3+CD25+ cells within the total CD4+ population or LLQ- AGI- or DYS-specific T cells. Error bars indicate median with IQR.
(TIF)

S3 Fig Proportion of CD4+ or TM+ T cells expressing FasL. (TIF)

S4 Fig Proportion of CD4+ or TM+ T cells expressing Tim-3. (TIF)

S5 Fig Gating strategy for flow cytometry analysis. Single cells were gated on (FSC-A vs FSC-H), then live CD14-CD19- cells, before identifying the lymphocyte population (FSC-A vs SSC-A). Of these CD3+CD4+ T cells gated on and within those HLA class II-peptide TM+ cells.
(TIF)

S1 Table Genes at least 2-fold up or down regulated in CMV-specific CD4+ T cells compared to EM CD4+ T cells. (XLSX)

S2 Table Genes differentially expressed between DYS-specific CD4+ T cells and EM CD4+ T cells. (XLSX)

S3 Table Genes at least 2-fold up or down regulated in LLQ-specific CD4+ T cells compared to EM CD4+ T cells. (XLSX)

## References

[1] Porichis F, Kaufmann DE (2011) HIV-specific CD4 T cells and immune control of viral replication. Curr Opin HIV AIDS 6: 174–180. 10.1097/COH.0b013e3283454058 21502921 PMC3265969

[2] Brown DM, Lee S, Garcia-Hernandez Mde L, Swain SL (2012) Multifunctional CD4 cells expressing gamma interferon and perforin mediate protection against lethal influenza virus infection. J Virol 86: 6792–6803. 10.1128/JVI.07172-11 22491469 PMC3393557

[3] Wilkinson TM, Li CK, Chui CS, Huang AK, Perkins M, Liebner JC, et al (2012) Preexisting influenza-specific CD4+ T cells correlate with disease protection against influenza challenge in humans. Nat Med 18: 274–280. 10.1038/nm.2612 22286307

[4] Zhou Y, Callendret B, Xu D, Brasky KM, Feng Z, Hensley LL, et al (2012) Dominance of the CD4(+) T helper cell response during acute resolving hepatitis A virus infection. J Exp Med 209: 1481–1492. 10.1084/jem.20111906 22753925 PMC3409494

[5] Altman JD, Moss PA, Goulder PJ, Barouch DH, McHeyzer-Williams MG, Bell JI, et al (1996) Phenotypic analysis of antigen-specific T lymphocytes. Science 274: 94–96. 8810254 10.1126/science.274.5284.94

[6] Gillespie GM, Wills MR, Appay V, O'Callaghan C, Murphy M, Smith N, et al (2000) Functional heterogeneity and high frequencies of cytomegalovirus-specific CD8(+) T lymphocytes in healthy seropositive donors. J Virol 74: 8140–8150. 10933725 10.1128/jvi.74.17.8140-8150.2000 PMC112348

[7] Khan N, Hislop A, Gudgeon N, Cobbold M, Khanna R, Nayak L, et al (2004) Herpesvirus-specific CD8 T cell immunity in old age: cytomegalovirus impairs the response to a coresident EBV infection. J Immunol 173: 7481–7489. 15585874 10.4049/jimmunol.173.12.7481

[8] Karrer U, Sierro S, Wagner M, Oxenius A, Hengel H, Koszinowski UH, et al (2003) Memory inflation: continuous accumulation of antiviral CD8+ T cells over time. J Immunol 170: 2022–2029. 12574372 10.4049/jimmunol.170.4.2022

[9] Noriega V, Redmann V, Gardner T, Tortorella D (2012) Diverse immune evasion strategies by human cytomegalovirus. Immunol Res 54: 140–151. 10.1007/s12026-012-8304-8 22454101

[10] Jackson SE, Mason GM, Wills MR (2011) Human cytomegalovirus immunity and immune evasion. Virus Res 157: 151–160. 10.1016/j.virusres.2010.10.031 21056604

[11] Hertoghs KM, Moerland PD, van Stijn A, Remmerswaal EB, Yong SL, van de Berg PJ, et al (2010) Molecular profiling of cytomegalovirus-induced human CD8+ T cell differentiation. J Clin Invest 120: 4077–4090. 10.1172/JCI42758 20921622 PMC2964975

[12] Komanduri KV, Feinberg J, Hutchins RK, Frame RD, Schmidt DK, Viswanathan MN, et al (2001) Loss of cytomegalovirus-specific CD4+ T cell responses in human immunodeficiency virus type 1-infected patients with high CD4+ T cell counts and recurrent retinitis. J Infect Dis 183: 1285–1289. 11262214 10.1086/319683

[13] Sabin CA, Phillips AN, Lee CA, Janossy G, Emery V, Griffiths PD (1995) The effect of CMV infection on progression of human immunodeficiency virus disease is a cohort of haemophilic men followed for up to 13 years from seroconversion. Epidemiol Infect 114: 361–372. 7705496 10.1017/s095026880005799x PMC2271271

[14] Einsele H, Roosnek E, Rufer N, Sinzger C, Riegler S, Loffler J, et al (2002) Infusion of cytomegalovirus (CMV)-specific T cells for the treatment of CMV infection not responding to antiviral chemotherapy. Blood 99: 3916–3922. 12010789 10.1182/blood.v99.11.3916

[15] Gamadia LE, Rentenaar RJ, van Lier RA, ten Berge IJ (2004) Properties of CD4(+) T cells in human cytomegalovirus infection. Hum Immunol 65: 486–492. 15172448 10.1016/j.humimm.2004.02.020

[16] Raeiszadeh M, Pachnio A, Begum J, Craddock C, Moss P, Chen FE (2015) Characterization of CMV-specific CD4+ T-cell reconstitution following stem cell transplantation through the use of HLA Class II-peptide tetramers identifies patients at high risk of recurrent CMV reactivation. Haematologica 100: e318–322. 10.3324/haematol.2015.123687 25975839 PMC5004434

[17] Walter EA, Greenberg PD, Gilbert MJ, Finch RJ, Watanabe KS, Thomas ED, et al (1995) Reconstitution of cellular immunity against cytomegalovirus in recipients of allogeneic bone marrow by transfer of T-cell clones from the donor. N Engl J Med 333: 1038–1044. 7675046 10.1056/NEJM199510193331603

[18] Tu W, Chen S, Sharp M, Dekker C, Manganello AM, Tongson EC, et al (2004) Persistent and selective deficiency of CD4+ T cell immunity to cytomegalovirus in immunocompetent young children. J Immunol 172: 3260–3267. 14978134 10.4049/jimmunol.172.5.3260

[19] Jonjic S, Mutter W, Weiland F, Reddehase MJ, Koszinowski UH (1989) Site-restricted persistent cytomegalovirus infection after selective long-term depletion of CD4+ T lymphocytes. J Exp Med 169: 1199–1212. 2564415 10.1084/jem.169.4.1199 PMC2189231

[20] Walton SM, Mandaric S, Torti N, Zimmermann A, Hengel H, Oxenius A (2011) Absence of cross-presenting cells in the salivary gland and viral immune evasion confine cytomegalovirus immune control to effector CD4 T cells. PLoS Pathog 7: e1002214 10.1371/journal.ppat.1002214 21901102 PMC3161985

[21] Sylwester AW, Mitchell BL, Edgar JB, Taormina C, Pelte C, Ruchti F, et al (2005) Broadly targeted human cytomegalovirus-specific CD4+ and CD8+ T cells dominate the memory compartments of exposed subjects. J Exp Med 202: 673–685. 16147978 10.1084/jem.20050882 PMC2212883

[22] Pourgheysari B, Khan N, Best D, Bruton R, Nayak L, Moss PA (2007) The cytomegalovirus-specific CD4+ T-cell response expands with age and markedly alters the CD4+ T-cell repertoire. J Virol 81: 7759–7765. 17409149 10.1128/JVI.01262-06 PMC1933343

[23] Casazza JP, Betts MR, Price DA, Precopio ML, Ruff LE, Brenchley JM, et al (2006) Acquisition of direct antiviral effector functions by CMV-specific CD4+ T lymphocytes with cellular maturation. J Exp Med 203: 2865–2877. 17158960 10.1084/jem.20052246 PMC2118179

[24] Fletcher JM, Vukmanovic-Stejic M, Dunne PJ, Birch KE, Cook JE, Jackson SE, et al (2005) Cytomegalovirus-specific CD4+ T cells in healthy carriers are continuously driven to replicative exhaustion. J Immunol 175: 8218–8225. 16339561 10.4049/jimmunol.175.12.8218

[25] Danke NA, Kwok WW (2003) HLA class II-restricted CD4+ T cell responses directed against influenza viral antigens postinfluenza vaccination. J Immunol 171: 3163–3169. 12960344 10.4049/jimmunol.171.6.3163

[26] Lucas M, Day CL, Wyer JR, Cunliffe SL, Loughry A, McMichael AJ, et al (2004) Ex vivo phenotype and frequency of influenza virus-specific CD4 memory T cells. J Virol 78: 7284–7287. 15194806 10.1128/JVI.78.13.7284-7287.2004 PMC421690

[27] Schulze Zur Wiesch J, Ciuffreda D, Lewis-Ximenez L, Kasprowicz V, Nolan BE, Streeck H, et al (2012) Broadly directed virus-specific CD4+ T cell responses are primed during acute hepatitis C infection, but rapidly disappear from human blood with viral persistence. J Exp Med 209: 61–75. 10.1084/jem.20100388 22213804 PMC3260872

[28] Ulsenheimer A, Lucas M, Seth NP, Tilman Gerlach J, Gruener NH, Loughry A, et al (2006) Transient immunological control during acute hepatitis C virus infection: ex vivo analysis of helper T-cell responses. J Viral Hepat 13: 708–714. 16970603 10.1111/j.1365-2893.2006.00747.x PMC4515975

[29] Scriba TJ, Zhang HT, Brown HL, Oxenius A, Tamm N, Fidler S, et al (2005) HIV-1-specific CD4+ T lymphocyte turnover and activation increase upon viral rebound. J Clin Invest 115: 443–450. 15668739 10.1172/JCI23084 PMC544605

[30] Long HM, Chagoury OL, Leese AM, Ryan GB, James E, Morton LT, et al (2013) MHC II tetramers visualize human CD4+ T cell responses to Epstein-Barr virus infection and demonstrate atypical kinetics of the nuclear antigen EBNA1 response. J Exp Med 210: 933–949. 10.1084/jem.20121437 23569328 PMC3646497

[31] Sester M, Sester U, Gartner B, Kubuschok B, Girndt M, Meyerhans A, et al (2002) Sustained high frequencies of specific CD4 T cells restricted to a single persistent virus. J Virol 76: 3748–3755. 11907214 10.1128/JVI.76.8.3748-3755.2002 PMC136081

[32] Appay V, van Lier RA, Sallusto F, Roederer M (2008) Phenotype and function of human T lymphocyte subsets: consensus and issues. Cytometry A 73: 975–983. 10.1002/cyto.a.20643 18785267

[33] Larbi A, Fulop T (2014) From "truly naive" to "exhausted senescent" T cells: when markers predict functionality. Cytometry A 85: 25–35. 10.1002/cyto.a.22351 24124072

[34] Pillai V, Ortega SB, Wang CK, Karandikar NJ (2007) Transient regulatory T-cells: a state attained by all activated human T-cells. Clin Immunol 123: 18–29. 17185041 10.1016/j.clim.2006.10.014 PMC1868523

[35] Kelwick R, Desanlis I, Wheeler GN, Edwards DR (2015) The ADAMTS (A Disintegrin and Metalloproteinase with Thrombospondin motifs) family. Genome Biol 16: 113 10.1186/s13059-015-0676-3 26025392 PMC4448532

[36] Saez-Borderias A, Guma M, Angulo A, Bellosillo B, Pende D, Lopez-Botet M (2006) Expression and function of NKG2D in CD4+ T cells specific for human cytomegalovirus. Eur J Immunol 36: 3198–3206. 17109473 10.1002/eji.200636682

[37] Day CL, Kaufmann DE, Kiepiela P, Brown JA, Moodley ES, Reddy S, et al (2006) PD-1 expression on HIV-specific T cells is associated with T-cell exhaustion and disease progression. Nature 443: 350–354. 16921384 10.1038/nature05115

[38] Radziewicz H, Ibegbu CC, Fernandez ML, Workowski KA, Obideen K, Wehbi M, et al (2007) Liver-infiltrating lymphocytes in chronic human hepatitis C virus infection display an exhausted phenotype with high levels of PD-1 and low levels of CD127 expression. J Virol 81: 2545–2553. 17182670 10.1128/JVI.02021-06 PMC1865979

[39] Duraiswamy J, Ibegbu CC, Masopust D, Miller JD, Araki K, Doho GH, et al (2011) Phenotype, function, and gene expression profiles of programmed death-1(hi) CD8 T cells in healthy human adults. J Immunol 186: 4200–4212. 10.4049/jimmunol.1001783 21383243 PMC3723805

[40] Savva GM, Pachnio A, Kaul B, Morgan K, Huppert FA, Brayne C, et al (2013) Cytomegalovirus infection is associated with increased mortality in the older population. Aging Cell 12: 381–387. 10.1111/acel.12059 23442093

[41] Simanek AM, Dowd JB, Pawelec G, Melzer D, Dutta A, Aiello AE (2011) Seropositivity to cytomegalovirus, inflammation, all-cause and cardiovascular disease-related mortality in the United States. PLoS One 6: e16103 10.1371/journal.pone.0016103 21379581 PMC3040745

[42] Day CL, Seth NP, Lucas M, Appel H, Gauthier L, Lauer GM, et al (2003) Ex vivo analysis of human memory CD4 T cells specific for hepatitis C virus using MHC class II tetramers. J Clin Invest 112: 831–842. 12975468 10.1172/JCI18509 PMC193667

[43] Roti M, Yang J, Berger D, Huston L, James EA, Kwok WW (2008) Healthy human subjects have CD4+ T cells directed against H5N1 influenza virus. J Immunol 180: 1758–1768. 18209073 10.4049/jimmunol.180.3.1758 PMC3373268

[44] Rentenaar RJ, Gamadia LE, van DerHoek N, van Diepen FN, Boom R, Weel JF, et al (2000) Development of virus-specific CD4(+) T cells during primary cytomegalovirus infection. J Clin Invest 105: 541–548. 10683384 10.1172/JCI8229 PMC289159

[45] Wunsch M, Zhang W, Hanson J, Caspell R, Karulin AY, Recks MS, et al (2015) Characterization of the HCMV-Specific CD4 T Cell Responses that Are Associated with Protective Immunity. Viruses 7: 4414–4437. 10.3390/v7082828 26258786 PMC4576189

[46] Swain SL, McKinstry KK, Strutt TM (2012) Expanding roles for CD4(+) T cells in immunity to viruses. Nat Rev Immunol 12: 136–148. 10.1038/nri3152 22266691 PMC3764486

[47] Carrasco J, Godelaine D, Van Pel A, Boon T, van der Bruggen P (2006) CD45RA on human CD8 T cells is sensitive to the time elapsed since the last antigenic stimulation. Blood 108: 2897–2905. 16857986 10.1182/blood-2005-11-007237

[48] Reeves MB, Sinclair JH (2013) Circulating dendritic cells isolated from healthy seropositive donors are sites of human cytomegalovirus reactivation in vivo. J Virol 87: 10660–10667. 10.1128/JVI.01539-13 23885077 PMC3807413

[49] van Leeuwen EM, Remmerswaal EB, Vossen MT, Rowshani AT, Wertheim-van Dillen PM, van Lier RA, et al (2004) Emergence of a CD4+CD28- granzyme B+, cytomegalovirus-specific T cell subset after recovery of primary cytomegalovirus infection. J Immunol 173: 1834–1841. 15265915 10.4049/jimmunol.173.3.1834

[50] Kwon B, Lee HW, Kwon BS (2002) New insights into the role of 4-1BB in immune responses: beyond CD8+ T cells. Trends Immunol 23: 378–380. 12133793 10.1016/s1471-4906(02)02263-9

[51] Jensen H, Folkersen L, Skov S (2012) Regulation and gene expression profiling of NKG2D positive human cytomegalovirus-primed CD4+ T-cells. PLoS One 7: e41577 10.1371/journal.pone.0041577 22870231 PMC3409864

[52] Groh V, Rhinehart R, Randolph-Habecker J, Topp MS, Riddell SR, Spies T (2001) Costimulation of CD8alphabeta T cells by NKG2D via engagement by MIC induced on virus-infected cells. Nat Immunol 2: 255–260. 11224526 10.1038/85321

[53] Zhu J, Yamane H, Paul WE (2010) Differentiation of effector CD4 T cell populations (*). Annu Rev Immunol 28: 445–489. 10.1146/annurev-immunol-030409-101212 20192806 PMC3502616

[54] Hegde NR, Dunn C, Lewinsohn DM, Jarvis MA, Nelson JA, Johnson DC (2005) Endogenous human cytomegalovirus gB is presented efficiently by MHC class II molecules to CD4+ CTL. J Exp Med 202: 1109–1119. 16216889 10.1084/jem.20050162 PMC2213219

[55] Pachnio A, Zuo J, Ryan GB, Begum J, Moss PA (2015) The Cellular Localization of Human Cytomegalovirus Glycoprotein Expression Greatly Influences the Frequency and Functional Phenotype of Specific CD4+ T Cell Responses. J Immunol 195: 3803–3815. 10.4049/jimmunol.1500696 26363059 PMC4592104

[56] Nishimura M, Umehara H, Nakayama T, Yoneda O, Hieshima K, Kakizaki M, et al (2002) Dual functions of fractalkine/CX3C ligand 1 in trafficking of perforin+/granzyme B+ cytotoxic effector lymphocytes that are defined by CX3CR1 expression. J Immunol 168: 6173–6180. 12055230 10.4049/jimmunol.168.12.6173

[57] Imai T, Hieshima K, Haskell C, Baba M, Nagira M, Nishimura M, et al (1997) Identification and molecular characterization of fractalkine receptor CX3CR1, which mediates both leukocyte migration and adhesion. Cell 91: 521–530. 9390561 10.1016/s0092-8674(00)80438-9

[58] Bolovan-Fritts CA, Spector SA (2008) Endothelial damage from cytomegalovirus-specific host immune response can be prevented by targeted disruption of fractalkine-CX3CR1 interaction. Blood 111: 175–182. 17895402 10.1182/blood-2007-08-107730 PMC2200803

[59] van de Berg PJ, Yong SL, Remmerswaal EB, van Lier RA, ten Berge IJ (2012) Cytomegalovirus-induced effector T cells cause endothelial cell damage. Clin Vaccine Immunol 19: 772–779. 10.1128/CVI.00011-12 22398244 PMC3346330

[60] Freeman RB Jr. (2009) The 'indirect' effects of cytomegalovirus infection. Am J Transplant 9: 2453–2458. 10.1111/j.1600-6143.2009.02824.x 19843027

[61] Morgan MD, Pachnio A, Begum J, Roberts D, Rasmussen N, Neil DA, et al (2011) CD4+CD28- T cell expansion in granulomatosis with polyangiitis (Wegener's) is driven by latent cytomegalovirus infection and is associated with an increased risk of infection and mortality. Arthritis Rheum 63: 2127–2137. 10.1002/art.30366 21437878

[62] Fuhrmann S, Streitz M, Reinke P, Volk HD, Kern F (2008) T cell response to the cytomegalovirus major capsid protein (UL86) is dominated by helper cells with a large polyfunctional component and diverse epitope recognition. J Infect Dis 197: 1455–1458. 10.1086/587692 18444801

[63] Kin NW, Sanders VM (2006) It takes nerve to tell T and B cells what to do. J Leukoc Biol 79: 1093–1104. 16531560 10.1189/jlb.1105625

[64] Kolmus K, Tavernier J, Gerlo S (2015) beta2-Adrenergic receptors in immunity and inflammation: stressing NF-kappaB. Brain Behav Immun 45: 297–310. 10.1016/j.bbi.2014.10.007 25459102

[65] Prosch S, Wendt CE, Reinke P, Priemer C, Oppert M, Kruger DH, et al (2000) A novel link between stress and human cytomegalovirus (HCMV) infection: sympathetic hyperactivity stimulates HCMV activation. Virology 272: 357–365. 10873779 10.1006/viro.2000.0367

[66] Dimitrov S, Lange T, Born J (2010) Selective mobilization of cytotoxic leukocytes by epinephrine. J Immunol 184: 503–511. 10.4049/jimmunol.0902189 19949113

[67] Irvine DJ, Purbhoo MA, Krogsgaard M, Davis MM (2002) Direct observation of ligand recognition by T cells. Nature 419: 845–849. 12397360 10.1038/nature01076

[68] Faroudi M, Utzny C, Salio M, Cerundolo V, Guiraud M, Muller S, et al (2003) Lytic versus stimulatory synapse in cytotoxic T lymphocyte/target cell interaction: manifestation of a dual activation threshold. Proc Natl Acad Sci U S A 100: 14145–14150. 14610278 10.1073/pnas.2334336100 PMC283560

[69] Purbhoo MA, Irvine DJ, Huppa JB, Davis MM (2004) T cell killing does not require the formation of a stable mature immunological synapse. Nat Immunol 5: 524–530. 15048111 10.1038/ni1058

[70] Tovar-Salazar A, Patterson-Bartlett J, Jesser R, Weinberg A (2010) Regulatory function of cytomegalovirus-specific CD4+CD27-CD28- T cells. Virology 398: 158–167. 10.1016/j.virol.2009.11.038 20034645 PMC2823847

[71] Honda T, Egen JG, Lammermann T, Kastenmuller W, Torabi-Parizi P, Germain RN (2014) Tuning of antigen sensitivity by T cell receptor-dependent negative feedback controls T cell effector function in inflamed tissues. Immunity 40: 235–247. 10.1016/j.immuni.2013.11.017 24440150 PMC4792276

[72] Bunce M, O'Neill CM, Barnardo MC, Krausa P, Browning MJ, Morris PJ, et al (1995) Phototyping: comprehensive DNA typing for HLA-A, B, C, DRB1, DRB3, DRB4, DRB5 & DQB1 by PCR with 144 primer mixes utilizing sequence-specific primers (PCR-SSP). Tissue Antigens 46: 355–367. 8838344 10.1111/j.1399-0039.1995.tb03127.x

[73] Elkington R, Shoukry NH, Walker S, Crough T, Fazou C, Kaur A, et al (2004) Cross-reactive recognition of human and primate cytomegalovirus sequences by human CD4 cytotoxic T lymphocytes specific for glycoprotein B and H. Eur J Immunol 34: 3216–3226. 15368271 10.1002/eji.200425203

[74] Slezak SL, Bettinotti M, Selleri S, Adams S, Marincola FM, Stroncek DF (2007) CMV pp65 and IE-1 T cell epitopes recognized by healthy subjects. J Transl Med 5: 17 17391521 10.1186/1479-5876-5-17 PMC1851947

[75] Khan N (2007) The immunological burden of human cytomegalovirus infection. Arch Immunol Ther Exp (Warsz) 55: 299–308. 18219760 10.1007/s00005-007-0037-3

[76] Roederer M, Nozzi JL, Nason MC (2011) SPICE: exploration and analysis of post-cytometric complex multivariate datasets. Cytometry A 79: 167–174. 10.1002/cyto.a.21015 21265010 PMC3072288

[77] Saeed AI, Sharov V, White J, Li J, Liang W, Bhagabati N, et al (2003) TM4: a free, open-source system for microarray data management and analysis. Biotechniques 34: 374–378. 12613259 10.2144/03342mt01

[78] Smyth GK (2004) Linear models and empirical bayes methods for assessing differential expression in microarray experiments. Stat Appl Genet Mol Biol 3: Article3 16646809 10.2202/1544-6115.1027

[79] Smyth GK, Speed T (2003) Normalization of cDNA microarray data. Methods 31: 265–273. 14597310 10.1016/s1046-2023(03)00155-5

[80] Ritchie ME, Silver J, Oshlack A, Holmes M, Diyagama D, Holloway A, et al (2007) A comparison of background correction methods for two-colour microarrays. Bioinformatics 23: 2700–2707. 17720982 10.1093/bioinformatics/btm412

[81] Huang da W, Sherman BT, Lempicki RA (2009) Systematic and integrative analysis of large gene lists using DAVID bioinformatics resources. Nat Protoc 4: 44–57. 10.1038/nprot.2008.211 19131956

[82] Huang da W, Sherman BT, Lempicki RA (2009) Bioinformatics enrichment tools: paths toward the comprehensive functional analysis of large gene lists. Nucleic Acids Res 37: 1–13. 10.1093/nar/gkn923 19033363 PMC2615629

