# Supplementary figures and images for: Cytomegalovirus Infection Leads to Development of High Frequencies of Cytotoxic Virus-Specific CD4+ T Cells Targeted to Vascular Endothelium
Source: PLoS Pathog. 2016 Sep 8;12(9):e1005832. doi: 10.1371/journal.ppat.1005832 (PMC5015996; doi:10.1371/journal.ppat.1005832)

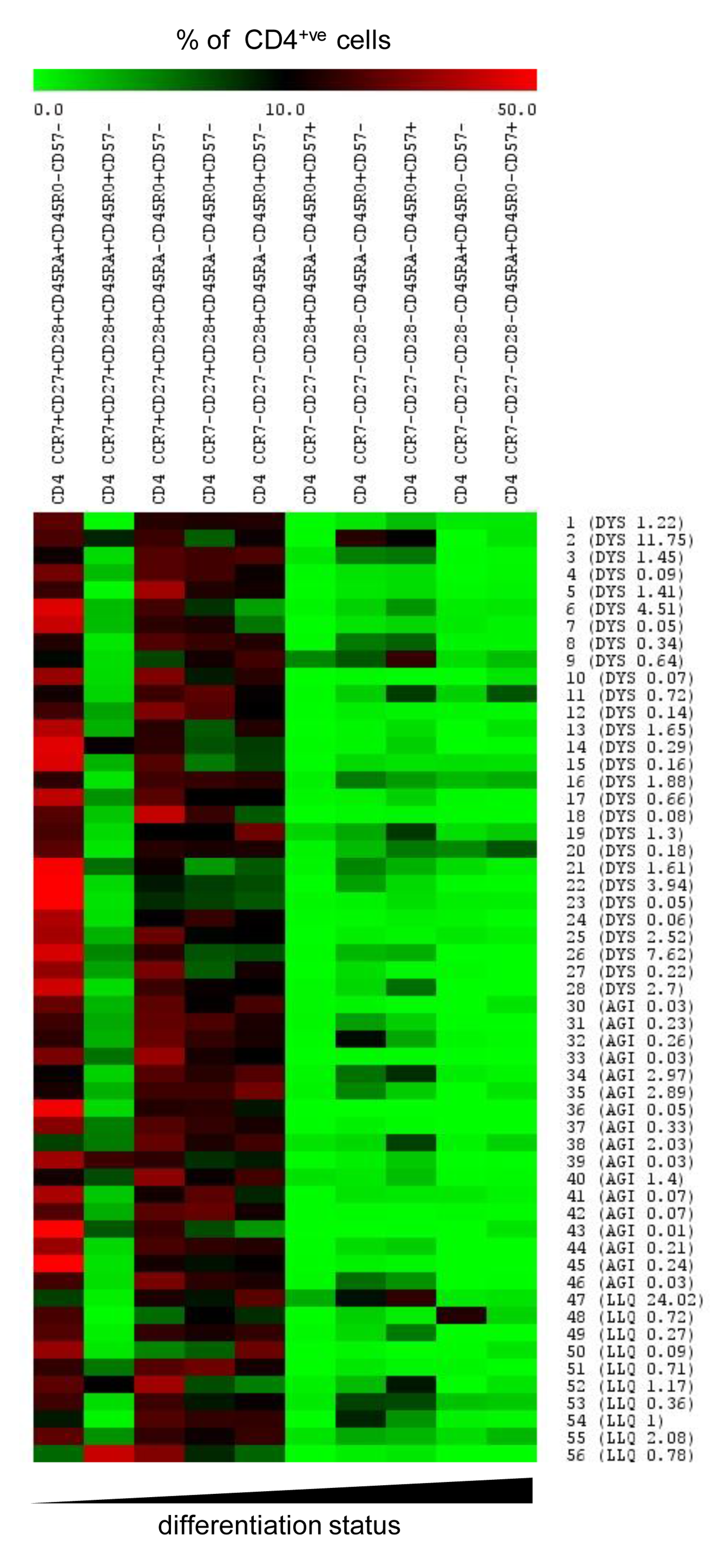

Supplement: S1 Fig — Cells were stained with CCR7, CD27, CD28, CD57, CD45RA and CD45RO to characterise the overall phenotypic profile. Using Boolean gating all possible combinations of the six surface markers were determined, and those subsets containing cells categorised according to differentiation status (increasing from left to right) as in Fig 3B. The heatmap is representing the proportion of CD4+ T cells within each subset for each of the donors studied. (TIF) [file ppat.1005832.s001.tif]

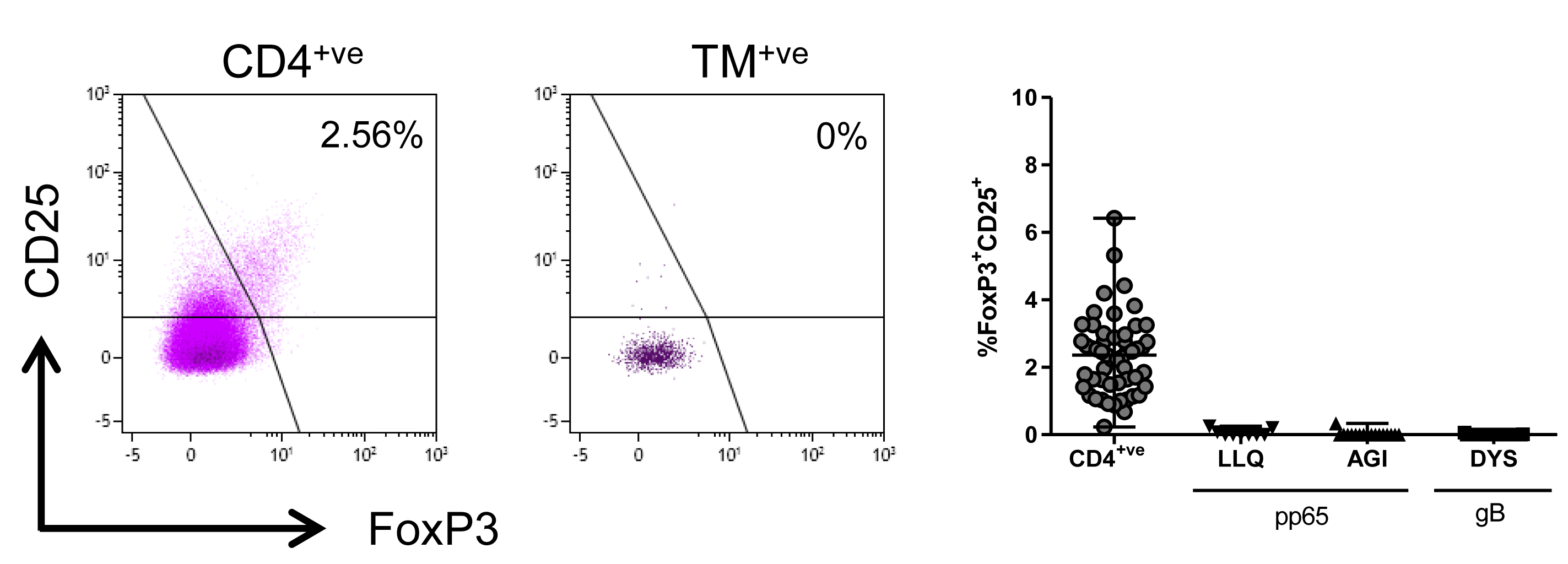

Supplement: S2 Fig — (A) Example scatter plots showing co-expression of FoxP3 and CD25 on CD4+ or TM+ T cells. (B) Frequency of FoxP3+CD25+ cells within the total CD4+ population or LLQ- AGI- or DYS-specific T cells. Error bars indicate median with IQR. (TIF) [file ppat.1005832.s002.tif]

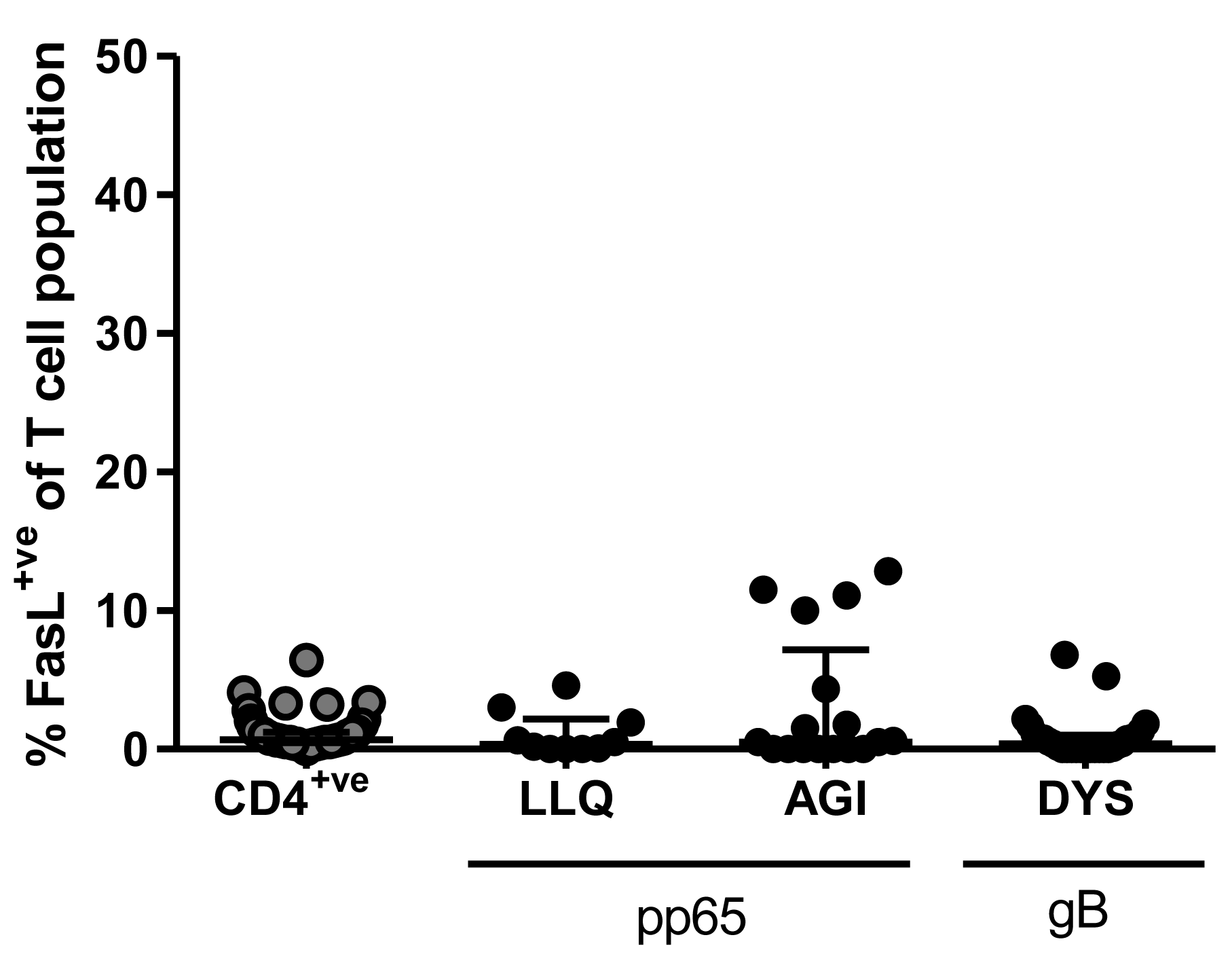

Supplement: S3 Fig — (TIF) [file ppat.1005832.s003.tif]

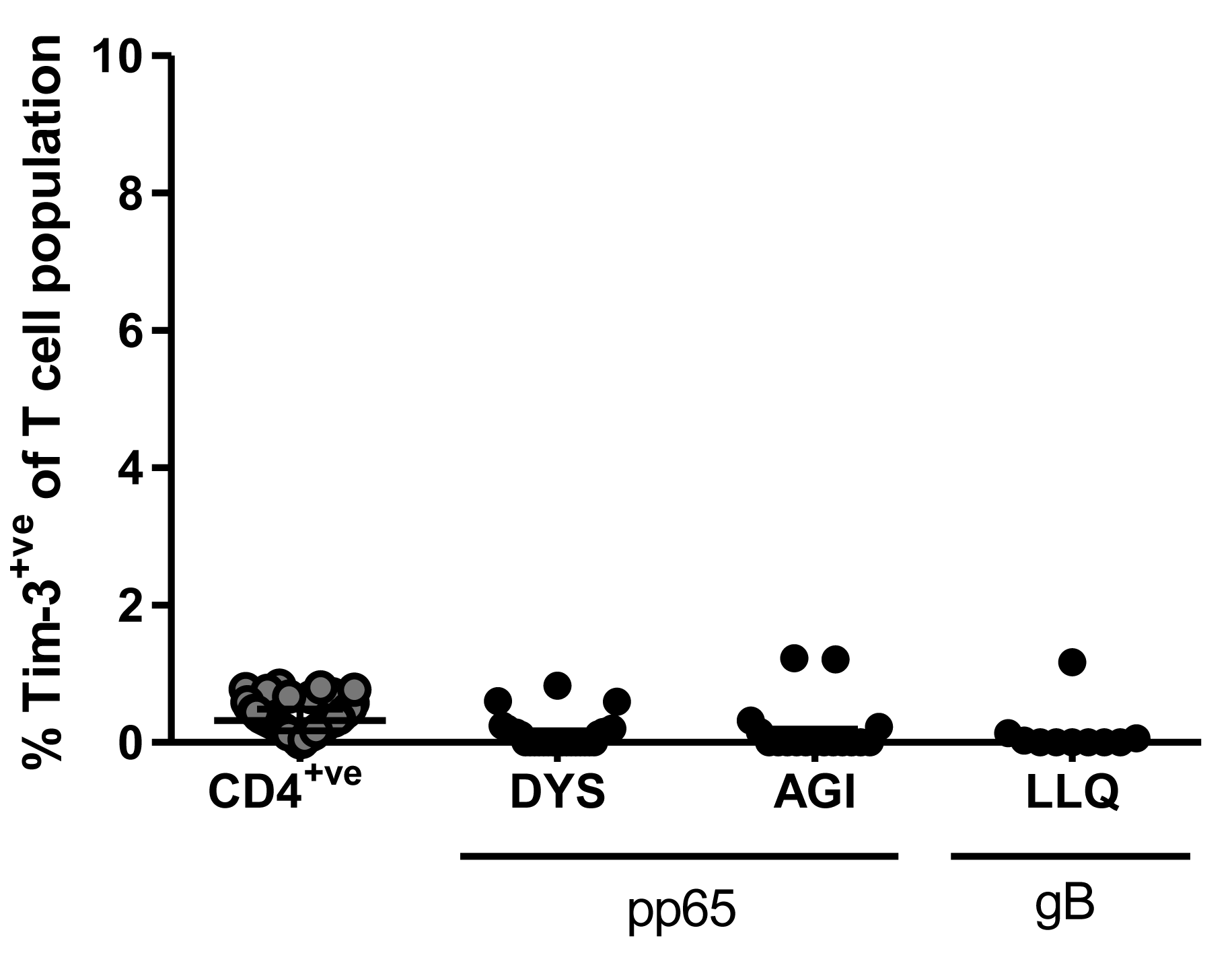

Supplement: S4 Fig — (TIF) [file ppat.1005832.s004.tif]

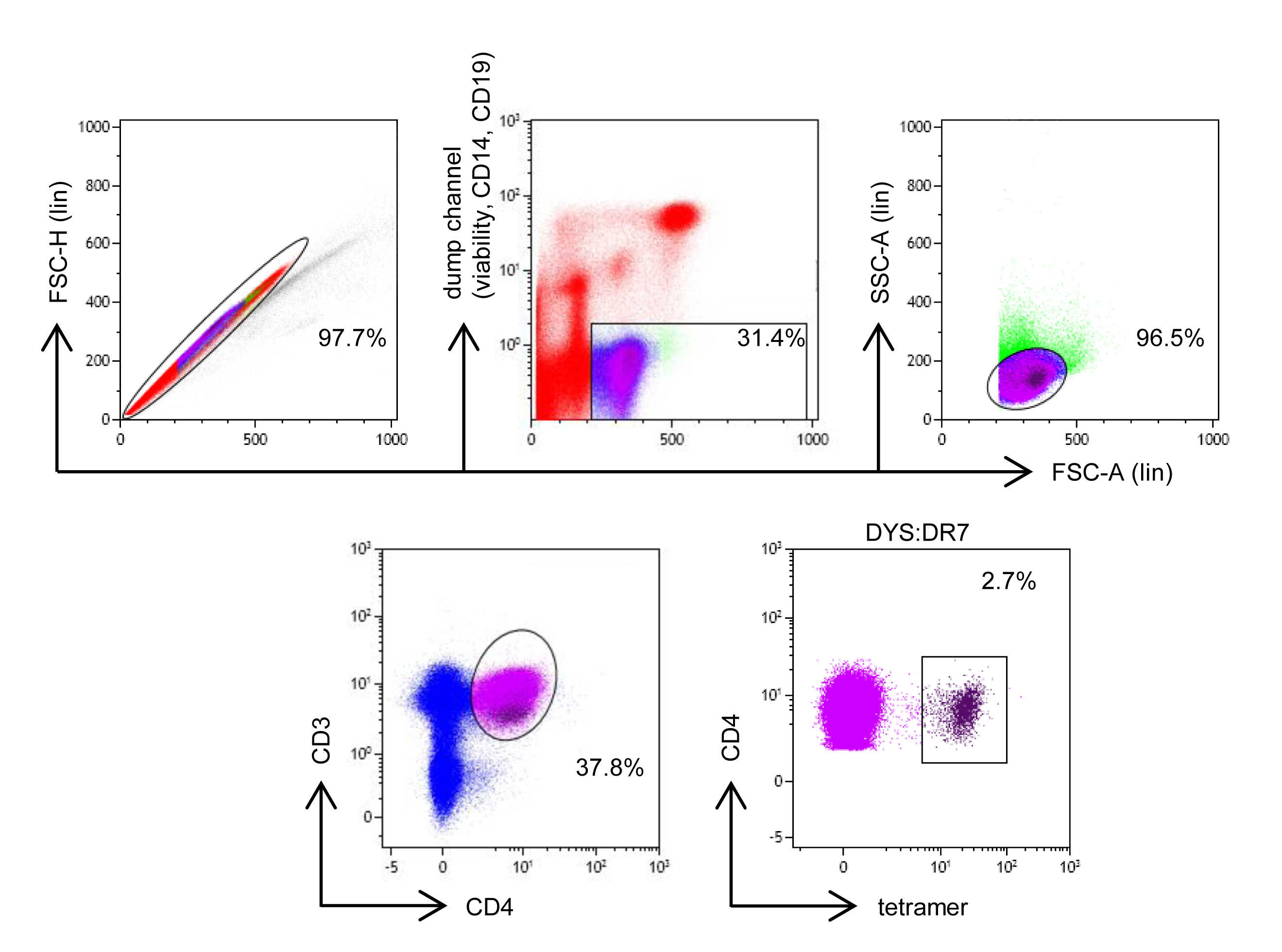

Supplement: S5 Fig — Single cells were gated on (FSC-A vs FSC-H), then live CD14-CD19- cells, before identifying the lymphocyte population (FSC-A vs SSC-A). Of these CD3+CD4+ T cells gated on and within those HLA class II-peptide TM+ cells. (TIF) [file ppat.1005832.s005.tif]
